# Biomimetic Second Coordination Sphere Effect within Cu-Peptoid Electrocatalyst Enables Homogeneous Water Oxidation at pH 7

**DOI:** 10.1021/acs.inorgchem.4c04501

**Published:** 2025-02-23

**Authors:** Guilin Ruan, Suraj Pahar, Natalia Fridman, Galia Maayan

**Affiliations:** Schulich Faculty of Chemistry, Technion−Israel Institute of Technology, Haifa 32000, Israel

## Abstract

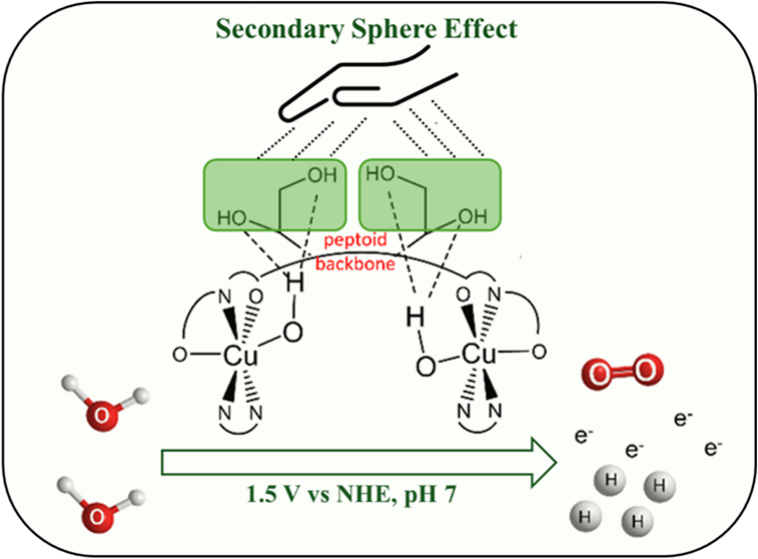

A dinuclear Cu-peptoid, Cu_2_(**BDiE**)_2_, having a diol side chain was developed as a homogeneous electrocatalyst for oxygen evolution reaction (OER) at neutral pH. The molecular structure of Cu_2_(**BDiE**)_2_ was characterized and concluded by ESI-MS, UV–vis, and single-crystal X-ray diffraction. Electrochemical, spectroscopic, and mechanistic studies revealed that borate buffer (the solution medium) has a minor effect during electrocatalysis; however, the diol side chain promotes a second coordination sphere effect via multiple hydrogen bonds which highly stabilize the complex, leading to an OER. Based on these observations and the collected data, we also suggest two different and unique mechanism pathways: a major one, which involves interactions of radical intermediates, which is buffer independent, and a minor one that resembles water nucleophilic attack (WNA) and is assisted by the borate buffer.

## Abstract 2

A dinuclear Cu-peptoid as a homogeneous electrocatalyst for water oxidation showcases an efficient secondary sphere effect on PECT and O−O bond formation processes, leading to electrocatalytic water oxidation at pH 7. Based on experimental results, two reaction pathways were identified: a unique major one, which is independent of the buffer, and a minor one that resembles a buffer-assisted WNA mechanism.

## 1 Introduction

Oxygen evolution reaction (OER) via water oxidation is the key challenge for producing green hydrogen fuel due to its high overpotential and slow kinetics.^1^ In nature, the solar-driven OER is catalyzed by the oxygen evolution complex (OEC) in the enzyme photosystem II near neutral pH, at pH 6.^2^ It was previously suggested that amino acid residues from the second coordination sphere of OEC, e.g., tyrosine and histidine, help stabilize its redox states and facilitate protons and electrons transfer processes,^3,4^ thus enabling high activity at near-neutral pH conditions, which is important for a good kinetic balance between OER and hydrogen evolution reaction.^5^ This motivates the design and development of biomimetic catalytic systems based on earth-abundant metals for OER at near-neutral pH.^6−8^

Various Cu complexes were developed as OER electrocatalysts, mostly at alkaline pH conditions,^9−11^ but their utilization for this reaction at neutral pH is still challenging.^12−18^ Several studies agreed that dinuclear Cu complexes are more reactive at pH 7 than mononuclear ones, due to their charge dispersion capability or synergistic effect,^13,19−21^ and a few others showed that redox-active ligands (first coordination sphere) participate in water oxidation.^22−25^ However, the utilization of second coordination sphere effects aimed to enable activity at pH 7 was not demonstrated. Peptoids, N-substituted glycine oligomers, represent an excellent platform for the design and development of second coordination sphere mimics of embedded catalytic centers,^26−30^ due to their efficient synthesis from primary amines, which allows high sequence versatility and tunability.^28,29,31^ Nevertheless, reported Cu-peptoid electrocatalysts for OER are only active at alkaline pH (9–11.5),^26,27^ and are not active at all at pH 7. To enable OER at pH 7 using a metallopeptoid-based electrocatalyst, we set to design a peptoid that incorporates, along with bipyridine (Bipy) and ethanol side chains for Cu(II) coordination, a diol group as the second coordination sphere mimic, for multiple hydrogen bonding stabilization of both the complex and its oxidized intermediates. Such stabilization is anticipated to mitigate the energy barrier and thus facilitate proton transfer processes within OER.^30^

In this study, we describe the design and synthesis of the peptoid **BDiE**, having bipyridine (Bpy), ethanol, and propanyl-diol groups as side chains, and its corresponding di-Cu-peptoid Cu_2_(**BDiE**)_2_ upon Cu(II) binding. We discovered that Cu_2_(**BDiE**)_2_ is a highly stable and active electrocatalyst OER at pH 7 and that the diol side chain is out of the first coordination sphere but crucial for stabilization of the metal site during electrolysis and for facilitating proton-coupled electron transfer (PCET) processes that lead to O–O bond formation.

## 2 Methods

### 2.1 Materials and Instrumentations

Materials and chemicals, Rink Amide resin, ethanolamine, 3-amino-1,2-propanediol, *N*,*N*′-diisopropylcarbodiimide (DIC), bromoacetic acid, 6-bromo-2,2′-bipyridine, and trifluoroacetic acid (TFA) were purchased from the same companies individually as reported previously from our lab.^26,27,32^ Reagents and solvents, dichloromethane, HPLC grade water, and acetonitrile were purchased from commercial sources and used without further purification, besides DMF that was dried with molecular sieves. 2-(2,2′-Bipyridine-6-yloxy)ethylamine was synthesized following a previous protocol.^33^ The −OH/diol group protection of ethanolamine/3-amino-1,2-propanediol was done by a reported procedure.^26^ Deionized water was obtained by a Milli-Q water purification system. A 0.2 M borate buffer solution (pH 7) was prepared using 0.2 M boric acid and adjusted by adding 0.2 M NaOH solution to achieve a final ionic strength of 0.2 M, which was monitored by an electronic pH meter.

The used instruments for purification and characterization of peptoid ligand and/or Cu-peptoid complex in this article, preparative HPLC, analytical HPLC, ESI and high-resolution-ESI mass spectrometry, UV–vis spectrophotometer, attenuated total reflection (ATR) spectrometer, Zeiss Ultra Plus high-resolution scanning electron microscope (SEM), energy-dispersive X-ray spectrometer (EDS), and Bruker NMR spectrometer AVIII400 have been reported with the same conditions as previously reported.^26,27,32,33^

### 2.2 Preparation and Characterization of Peptoid Oligomers

The peptoids **BDiE** were synthesized manually on Rink amide resin using the submonomer approach.^34^ Typically, this approach includes two iterated steps after the deprotection of the resin by 20% piperidine DMF solution for 20 min, bromoacetylation, and amine displacement reactions. In each cycle, once bromoacetic acid is incorporated into the resin, the sequential amines are added in the following step for displacing the bromide via SN2 reactions. For peptoid **BDiE**, 2-(2,2′-bipyridine-6-yloxy)ethylamine, protected 3-amino-1,2-propanediol, and protected ethanolamine were subsequently introduced into the resin in each iterating cycle, with reaction times of 5 h, 20 min, and 20 min, respectively. Following the end of the iteration, the peptoid was cleaved from the resin by 95% TFA in water (40 mL/g resin) for 20 min. The cleavage solution was then dried in a vacuum, and then the residue solid (or gel) was redissolved in HPLC solvent (1:1 HPLC grade acetonitrile/water) and lyophilized overnight. More details of peptoid synthesis have been reported previously.^27,33^ The peptoid **BDiE** was further purified to >95% by preparative HPLC and lyophilized overnight, obtaining a yield of 40–50%. The peptoids after purification were characterized by analytical HPLC, ESI-MS, and ^1^H NMR (Figures S1, S2, and S19).

### 2.3 Synthesis of Complex Cu_2_(**BDiE**)_2_

Once the peptoid ligand was prepared, 0.1 mmol of it was dissolved in 1 mL of *n*-propanol, and then this solution was treated with 0.1 mmol of copper perchlorate hexahydrate and stirred for 4 h. A greenish-blue solid precipitate was obtained and further isolated by centrifugation, washed 3 times with *n*-propanol, and dried in a vacuum. The solid product yielded about 75%. Then the solid compound was redissolved in water in an NMR tube and left in open air. A few days or a week later, a blue crystal was obtained. The isolated crystal was then analyzed in single-crystal X-ray diffraction, ESI-MS, UV–vis, EPR, and bond valence sum (BVS) calculation (Figures S3, S4, S20, S21 and Table S1). More details of the obtained crystal structure are accessed by the Cambridge Crystallographic Data Centre (CCDC number: 2411148).

Note: copper perchlorate hexahydrate has significant safety hazards, including its potential to cause fires or explosions upon contact with reducing agents or combustible substances. It should be tackled with caution in a properly ventilated fume hood, using proper personal protective equipment, including gloves, goggles, and a lab coat.

### 2.4 Electrochemical Methods

The electrochemical properties of the prepared Cu complex were measured using an IVIUMSTAT.XRe or PalmSens potentiostat/galvanostat. The Cu complex was initially characterized at RT by cyclic voltammetry (CV), as well as differential pulse voltammetry (DPV), to understand the working potential. These measurements were carried out in a beaker cell with a three-electrode system. Glassy carbon (GC) was used as a working electrode (WE) (0.07 cm^2^) unless mentioned specifically otherwise, Ag/AgCl as a reference electrode, and Pt as a counter electrode. The controlled potential electrolysis (CPE) experiment according to applied potential was done to evaluate the Faradaic efficiency (FE%), as well as electrochemical stability. Oxygen evolution was monitored in the gas phase with a Fixed-Needle-Type Oxygen Minisensor (from PyroScience) placed in the headspace of the reaction vassal (working electrode side). The Faraday efficiency was calculated by the total charge passed during CPE and the total amount of generated oxygen as a four-electron oxidation process: FE% = *n*(O_2_, exp.)/(*Q*/*nF*) × 100%, where *n*(O_2_, exp.) is the measured oxygen by the sensor from the CPE experiment, mol; *Q* is the accumulated charge from the CPE experiment, C; *n* is the number of electrons transferred, which is 4; and *F* is the Faraday constant, which is 96,485 C/mol. All reported potentials in the present work are reported vs NHE by adding 0.20 V to the measured potential unless specified otherwise. The spectroelectrochemistry experiment was done in a special cuvette where 1 mL of catalyst solution was placed and it contained three electrode chambers: Pt mesh as working, Pt as counter, and Ag/AgCl as reference electrodes, and spectral were recorded in Agilent Technologies Cary 60 UV–vis spectrophotometer.

## 3 Results and Discussion

### 3.1 Synthesis and Analysis of Molecular Structure

**Figure 1:**
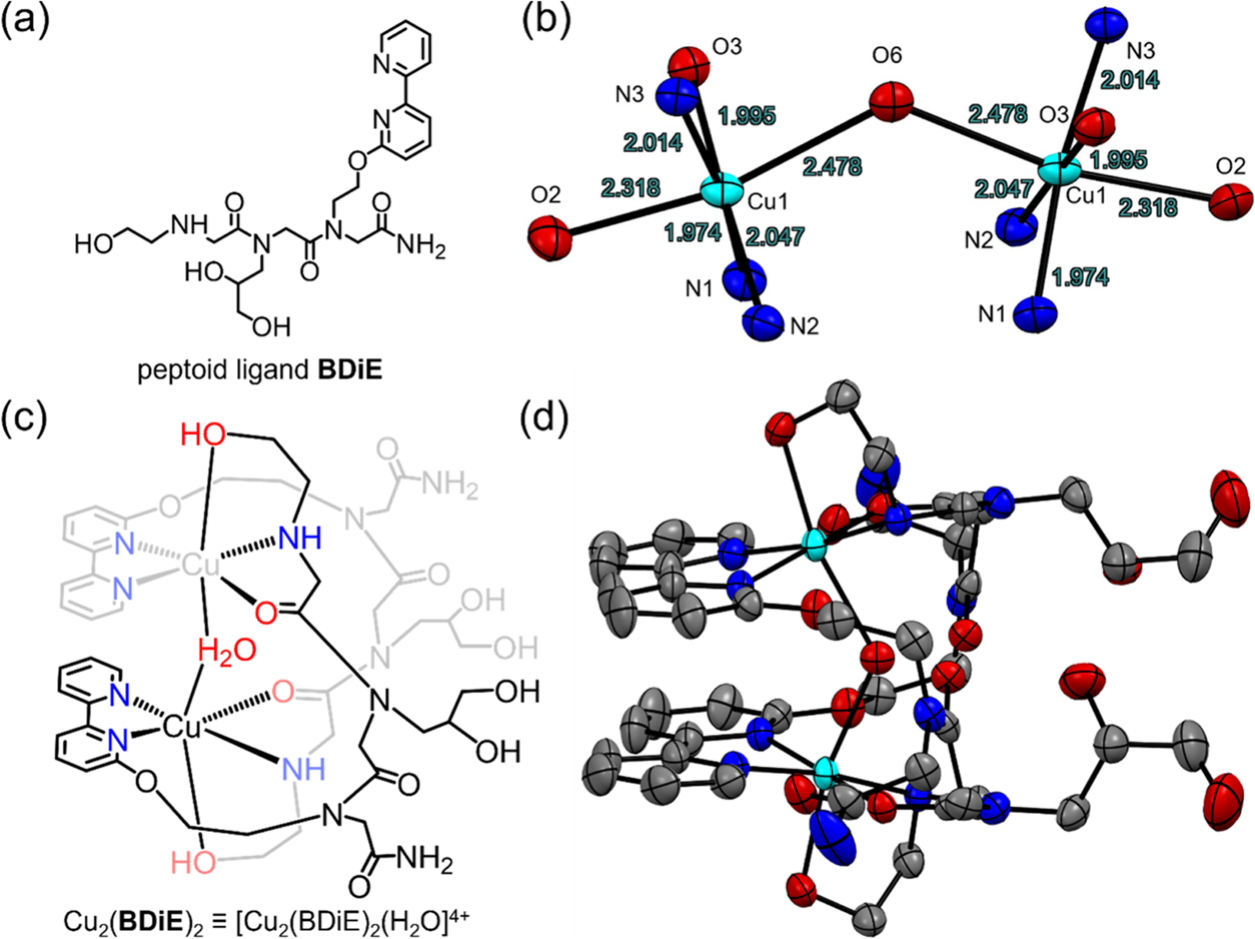
(a) Molecular design of ligand **BDiE**; (b) dinuclear Cu centers with atom and bond distance in the crystal structure (ellipsoid style of 50% probability level) of Cu_2_(**BDiE**)_2_; (c, d) molecular and crystal structures of Cu_2_(**BDiE**)_2_, hydrogen atoms, and guest molecules are excluded to enhance clarity.

The peptoid **BDiE** (Figure 1a) was synthesized on a solid support, cleaved, and purified by high-performance liquid chromatography to >99% purity (Figure S1).^34^ Its molecular weight, determined by ESI-MS, was consistent with the expected mass for its sequence (Figure S2). **BDiE** was treated with 1 equiv of [Cu(H_2_O)_6_](ClO_4_)_2_ in *n*-propanol, forming a greenish-blue precipitate, which was isolated and recrystallized from water and characterized by single-crystal X-ray analysis (Figure 1b). The bond valence sum (BVS) calculation from the bond lengths was obtained as 2.02 for both Cu ions, indicating +2 oxidation state (Table S1).^35^ Therefore, the obtained complex was [Cu_2_(BDiE)_2_(H_2_O)](ClO_4_)_4_ (abbreviated as Cu_2_(**BDiE**)_2_). The distance between two Cu ions is 4.467 Å and they are bridged by a H_2_O molecule with a Cu–O–Cu angle of 128.65° and a symmetric Cu–O bond length of 2.478 Å (Figure 1). Each Cu is coordinated to three N atoms (two from Bpy of one peptoid and one from the secondary amine of the other peptoid) and three O atoms (one from the H_2_O bridge, one from the −OH side chain, and 1 from a backbone carbonyl). The ESI-MS spectrum (Figure S3) showcases a dominant *m*/*z* peak of 1433.2173 consistent with [Cu_2_(BDiE)_2_(ClO_4_)_3_]^+^, suggesting that the complex exists as the dinuclear form also in the liquid phase and that the H_2_O bridge is labile.^27^ In addition, using UV–vis spectroscopy, the absorbance band at 323 nm assigned to Cu_2_(**BDiE**)_2_ increased linearly with the increase in the concentration of the complex in borate buffer at pH 7 (Figure S4), indicating that Cu_2_(**BDiE**)_2_ is maintained as one single species in these conditions, which is stable for at least 24 h. The EPR spectrum of Cu_2_(**BDiE**)_2_ showed a signal at half-field region (ΔMS = ±2, Figure S20), further supporting that it is a dinuclear complex.^36,37^

### 3.2 Electrochemical Properties and Oxygen Evolution

**Figure 2:**
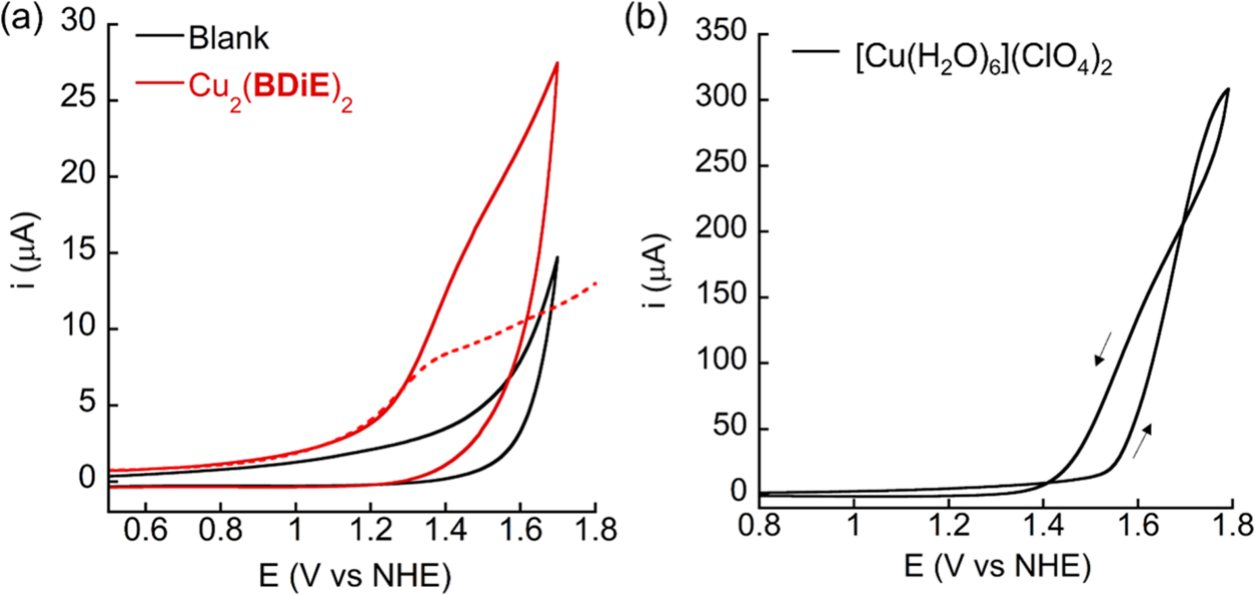
(a) CV with and without 0.5 mM Cu_2_(**BDiE**)_2_; DPV of Cu_2_(**BDiE**)_2_ is shown as a dashed line; (b) CV with 1 mM [Cu(H_2_O)_6_](ClO_4_)_2_ salt; the arrows show the direction of scanning; all experiments were performed in 0.2 M borate buffer at pH 7 with a scan rate of 100 mV/s.

The electrochemical properties of Cu_2_(**BDiE**)_2_ were measured in 0.2 M borate buffer at pH 7. Cyclic voltammetry (CV) and differential pulse voltammetry (DPV) were obtained using glassy carbon (GC) as a working electrode (WE), Ag/AgCl as a reference electrode, and platinum wire as a counter electrode. An oxidation wave with high current intensity was displayed in the CV measurement of Cu_2_(**BDiE**)_2_ at *E*_p_ = +1.37 V vs NHE (defined as peak potential value in the corresponding DPV, Figure 2a). Together with a reversed CV scan for the detection of O_2_ from the reduction at −0.2 V (vs NHE, unless specified otherwise), where the reduction is defined as Cu^II^/Cu^I^ and it can further reduce oxygen, this oxidation *E*_p_ is confirmed to be a catalytic event of OER (Figure S5). CV of [Cu(H_2_O)_6_](ClO_4_)_2_ in the same buffer resulted in a crossover indicating heterogeneous catalysis behavior (Figure 2b). These distinct results between Cu_2_(**BDiE**)_2_ and [Cu(H_2_O)_6_](ClO_4_)_2_ indicate that free Cu ion impurity does not exist in the solution of Cu_2_(**BDiE**)_2_. Therefore, the catalytic activity is attributed to added Cu_2_(**BDiE**)_2_.

**Figure 3:**
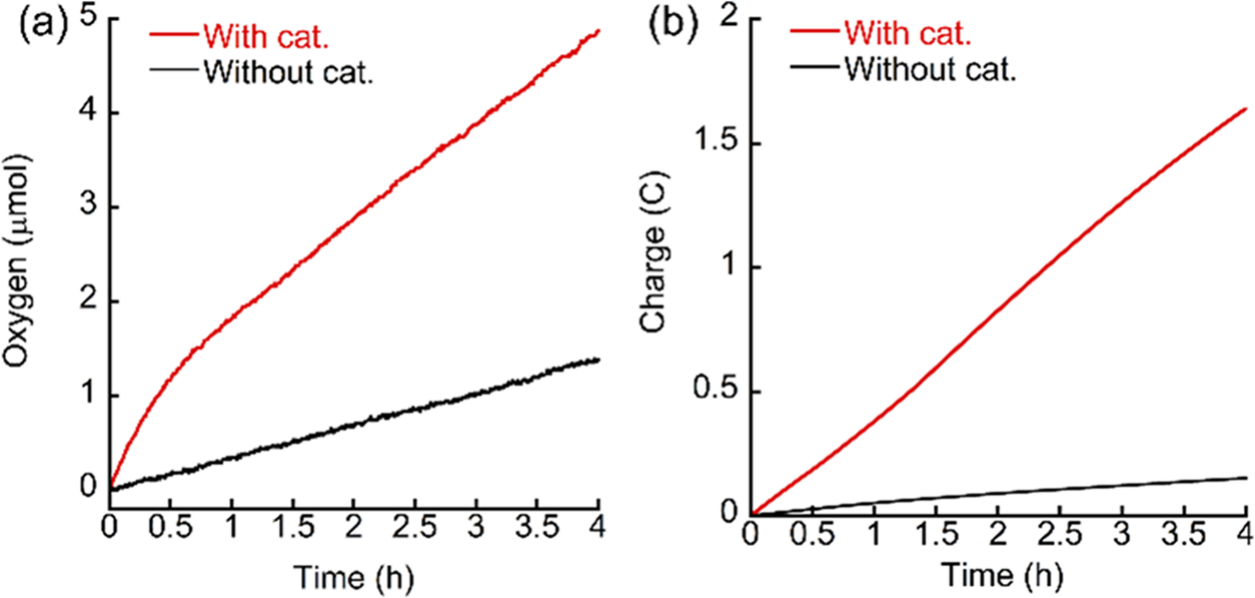
(a) O_2_ evolution and (b) charge accumulation during 4 h CPE with and without Cu_2_(**BDiE**)_2_ (0.25 mM). The experiments were conducted at +1.5 V using ITO as the working electrode, Ag/AgCl as the reference electrode, and Pt wire as the counter electrode in an H-cell.

**Figure 4:**
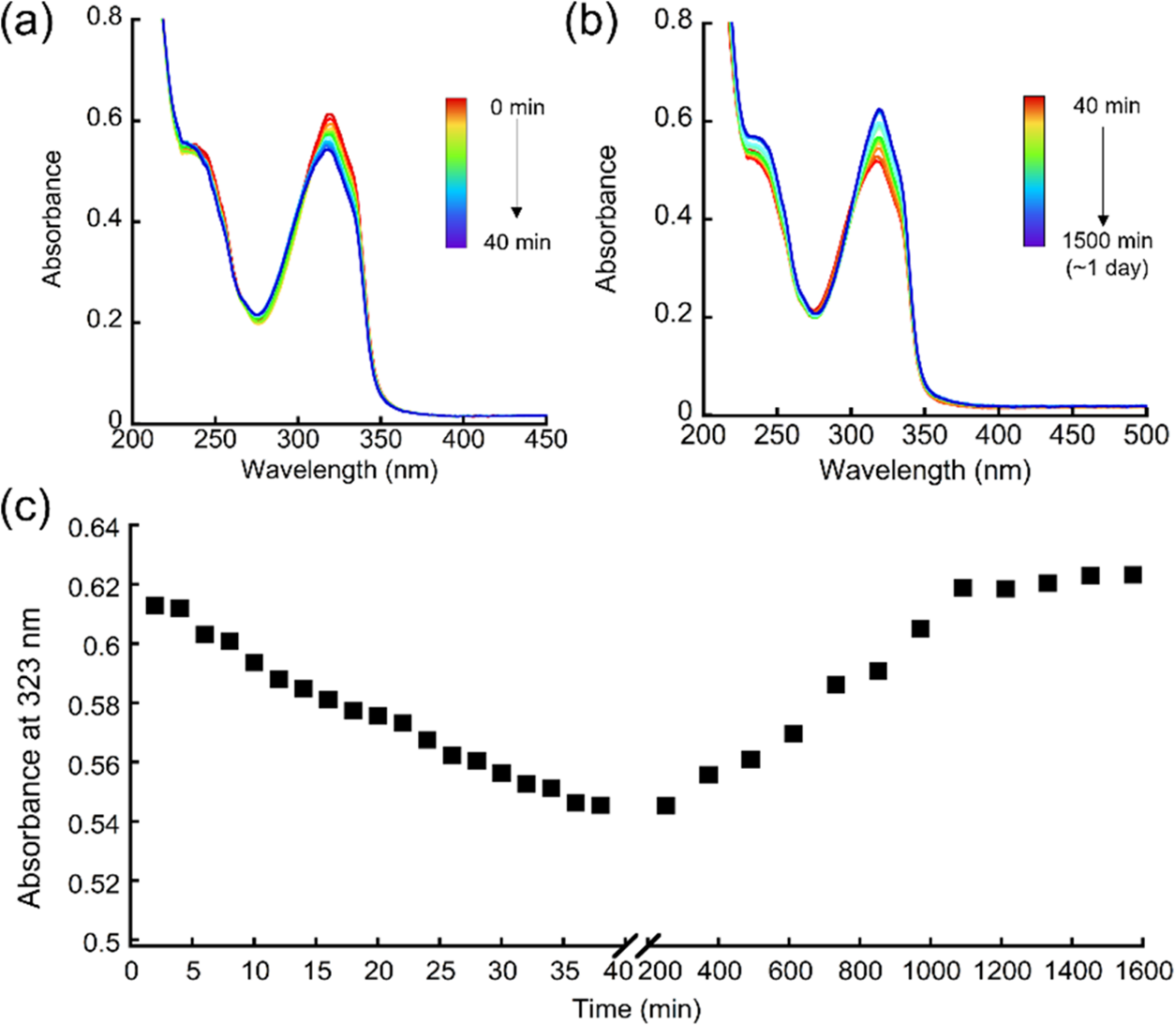
(a) UV–vis spectra during the 40 min spectroelectrochemical experiment with applied potential +1.5 V using Pt net as WE, Ag/AgCl as RE, and Pt wire as counter electrode in 0.2 M borate buffer at pH 7; (b) consecutive UV–vis spectra after 40 min spectroelectrochemical experiment when three electrodes were removed; (c) absorbance plot at 323 nm vs time (min).

O_2_ evolution was investigated using a controlled potential electrolysis (CPE) experiment at +1.5 V with ITO as WE in 0.2 M borate buffer at pH 7 (Figure 3). In 4 h, the oxygen increment was 4.9 μmol with 0.25 mM of Cu_2_(**BDiE**)_2_ and 1.3 μmol without it. It resulted in a turnover number (TON) of 3, proving that oxygen evolution was produced in an electrocatalytic behavior instead of a stoichiometric reaction. This electrolysis afforded a charge accumulation of 1.6 or 0.1 C in the presence or absence of Cu_2_(**BDiE**)_2_. Based on 4e^–^ transfer water oxidation, the Faradaic efficiency (FE%) was calculated to be 85%. As OER can last for 4 h, we expected that Cu_2_(**BDiE**)_2_ is stable in these conditions. Indeed, a 40 min CPE spectroelectrochemical study (Figure 4) showed that when OER started, the absorbance at 323 nm started to decrease. At the end of the reaction, the UV–vis spectrum was measured again after the three electrodes were removed from the solution, and it showed that the reduced absorbance recovered to the original level within 20 h, with an isosbestic point near 303 nm. The long time of absorbance recovery might imply the high stability of intermediates, indicating that peptoid ligand assists the stabilization of high oxidation state species.^26^

### 3.3 Homogeneity and Kinetics Studies

Additionally, ITO WE from the 4 h CPE experiment was examined with HR-SEM and EDS before and after the 4 h OER (Figures S8 and S9). The results showed that no particles were found on the electrode’s surface in HR-SEM, and no signal assigned as Cu element was found attached during EDS after electrolysis. A common concern of homogeneity is that the dissolved catalyst may form in situ solid oxide on WE during electrocatalysis and redissolve back into solution after the applied potential is stopped. This phenomenon might be detected on the surface of WE. However, since the absorbance at 323 nm defined as Cu_2_(**BDiE**)_2_ recovered back to the same level after electrocatalysis without placing WE in the solution, the absorbance change is by homogeneous species and there is no Cu ion loss in the solution. Therefore, this concern can be eliminated. Moreover, the CV scans at different scan rates (Figure S10) showed that the noncatalytic peak current *i*_d_ from Cu^II/I^ oxidation of Cu_2_(**BDiE**)_2_ varies linearly with the square root of the scan rate, ν^1/2^. Considering the irreversibility, *i*_d_, and ν^1/2^ follow the below relation
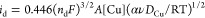
1

The value of the diffusion coefficient *D*_Cu_ was calculated to be 9 × 10^–6^ cm^2^/s, fitting in the diffusion-controlled process.^38^ Furthermore, CVs before and after the 4 h CPE are identical without any drastic change (Figure S11). Collectively, they indicate that the catalytic process is truly homogeneous.

CVs at different scan rates and concentrations of Cu_2_(**BDiE**)_2_ were performed to gain insights into the kinetics (Figure S12). The linear dependence of the catalytic current *i*_cat_ on the concentration of Cu_2_(**BDiE**)_2_ points out that the kinetics of OER is performed by single-molecule catalysis with first-order kinetics. Therefore, the catalytic process obeys the relationship displayed in eq 2
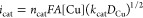
2

The correlation and slope value between *i*_cat_/*i*_d_ and ν^–1/2^ is obtained by the division of eqs 1 and 2
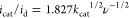
3

Subsequently, TOF (*k*_cat_) is calculated to be 0.2 s^–1^ by the linear slope value.^9^ TOF was also determined by foot-of-the-wave analysis (FOWA) as 76 s^–1^ (Figure S12). Although Cu_2_(**BDiE**)_2_ follows an activity–stability trade-off,^39^ the high stability should lead to a higher amount of oxygen over time, thus compensating, in the long run, for the moderate kinetics.

### 3.4 Role of Borate Buffer

**Figure 5:**
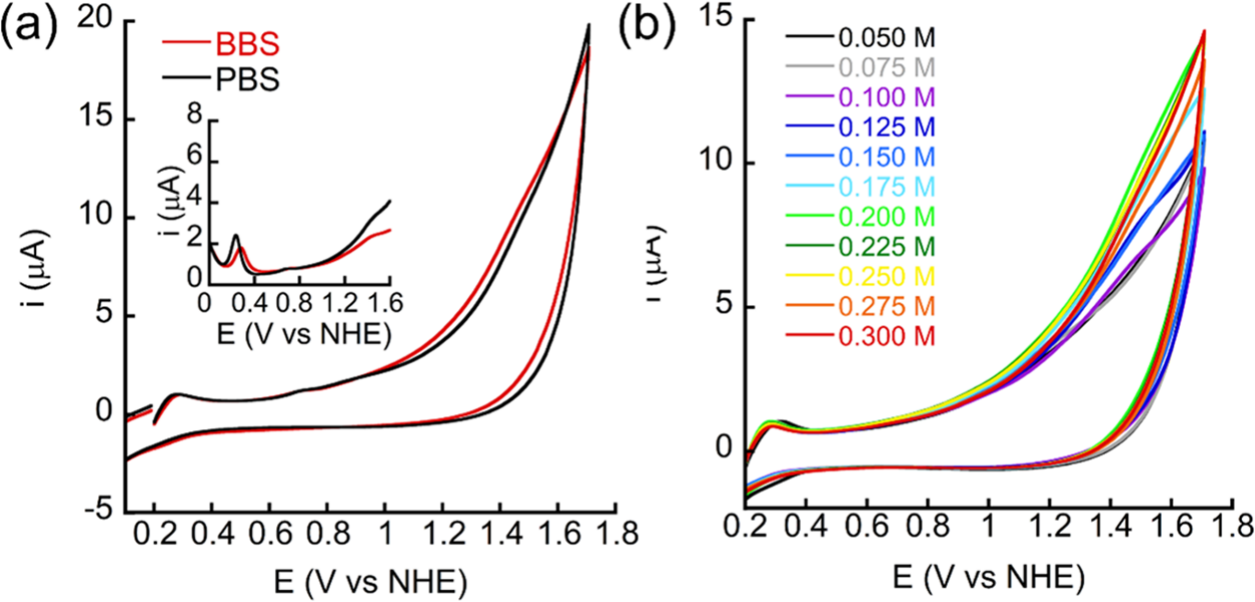
(a) CVs and DPVs of 0.4 mM Cu_2_(**BDiE**)_2_ in 0.2 M borate/phosphate buffer at pH 7; (b) CVs of 0.25 mM Cu_2_(**BDiE**)_2_ in different concentrations of borate buffer.

We anticipated that the borate buffer does not have a major role in OER in these conditions because the main species of the buffer at pH 7 is the neutral B(OH)_3_ rather than the charged B(OH)_4_^–^, which is present in pH > 9.2,^27^ and B(OH)_3_ is not capable of a nucleophilic attack on the metal-oxo intermediate for O–O bond formation,^40^ which is a rate-determining step for OER. Indeed, CVs and DPVs with Cu_2_(**BDiE**)_2_ done in borate and phosphate buffers displayed similar oxidation potential and current intensity (Figure 5a), indicating that the borate buffer is not superior to phosphate buffer in these conditions, probably contributing to transfer proton instead of critically transfer oxygen-atom.^41^ Furthermore, the kinetic value *k*_B_, assigning the partial rate of OER determined by the B(OH)_3_, was calculated as 0.88 M^–1^ s^–1^ (Figure 5b and Table S2), which is similar to that of H_2_O at pH 7,^41^ and about 900 times slower than that of B(OH)_4_^–^ at pH > 9, indicating that B(OH)_3_ has no superior role over H_2_O (as a proton acceptor) for OER in these conditions.

### 3.5 Mechanistic Study

**Figure 6:**
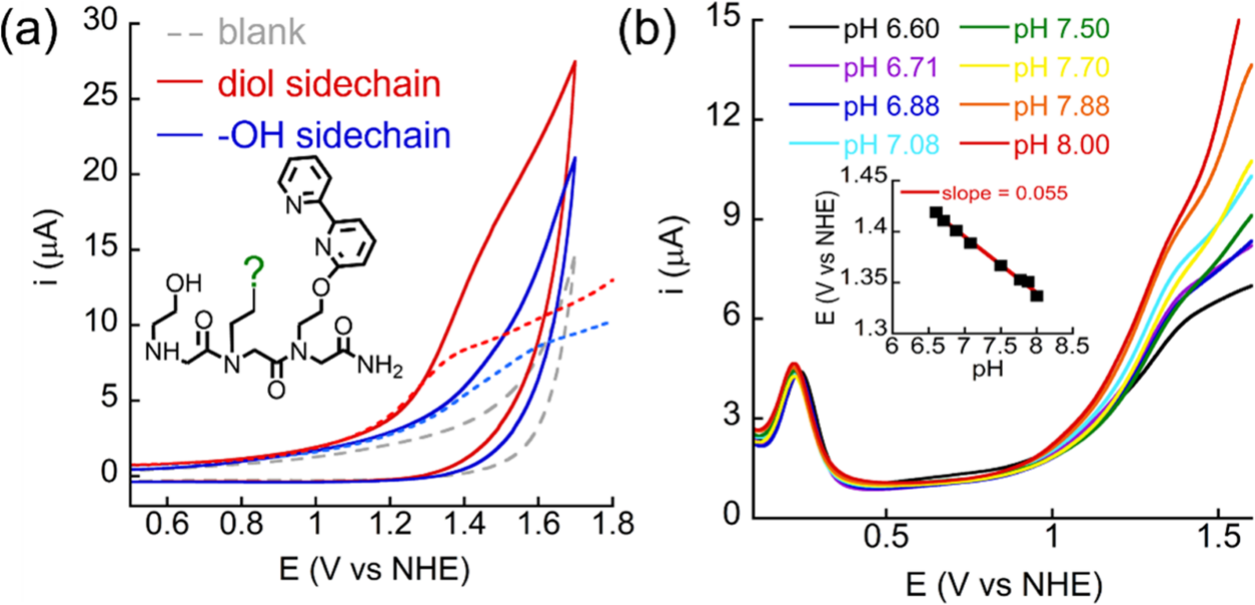
(a) CVs with Cu_2_(**BDiE**)_2_ and reported catalyst^17^ and without catalyst in 0.2 M borate buffer at pH 7; (b) pH study using Cu_2_(**BDiE**)_2_ in 0.2 M borate buffer, inset is the plotted Pourbaix diagram.

**Figure 7:**
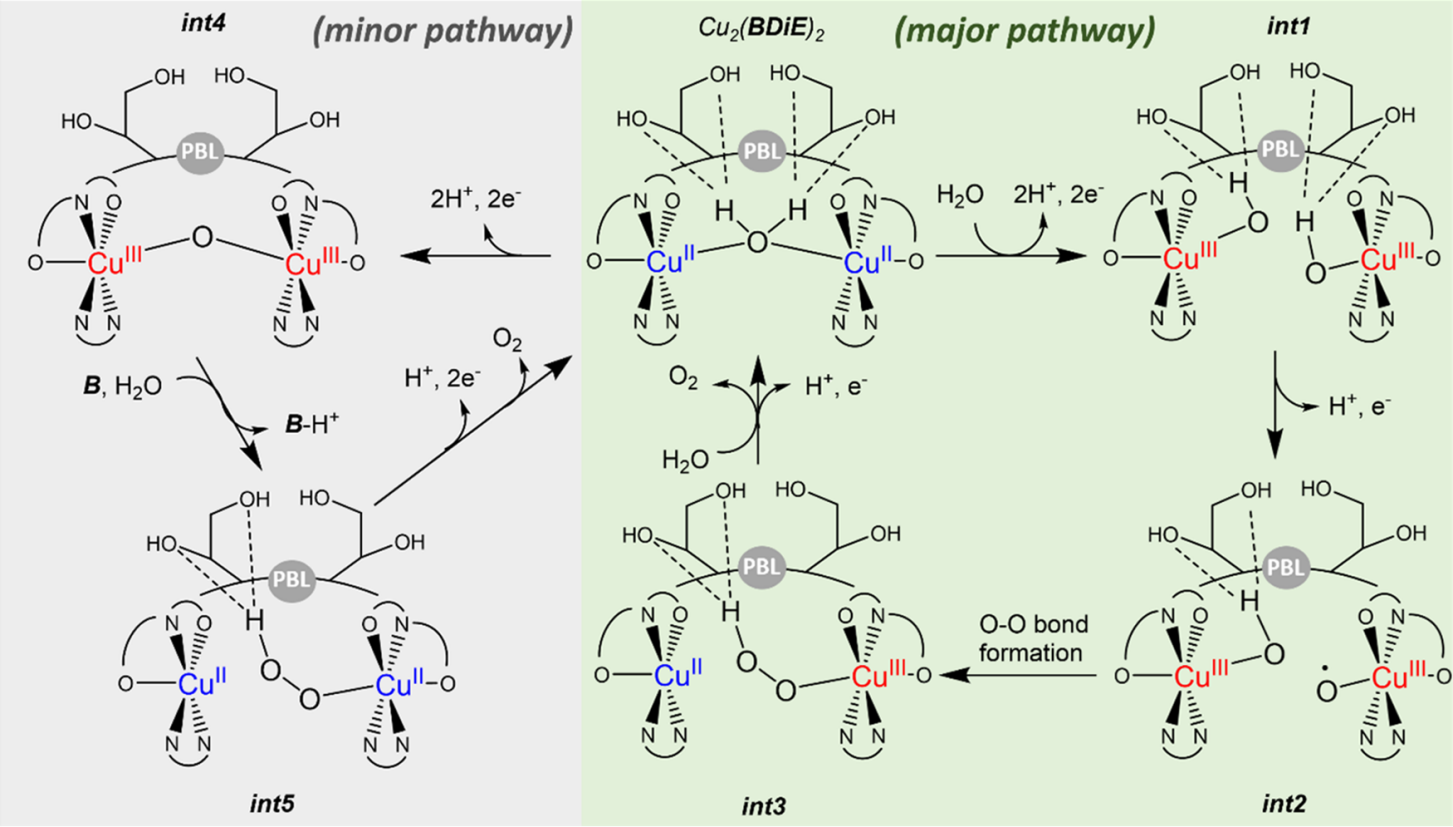
Proposed mechanism of using Cu_2_(**BDiE**)_2_ for homogeneous electrocatalytic OER at pH 7. PBL: peptoid backbone linker, B: borate buffer species.

In the lack of a major buffer effect on the catalytic OER, we suggest that the diol side chain in **BDiE**, which is in the second coordination sphere of the metal centers within Cu_2_(**BDiE**)_2_, has an important role in the catalytic activity at pH 7. First, a similar dinuclear Cu-peptoid having only one −OH group on the dangling side chain^27^ showed lower catalytic activity and higher onset potential compared with those of Cu_2_(**BDiE**)_2_ (Figures 6a and S14). Second, to further understand the role of the diol side chain, the OER mechanism using Cu_2_(**BDiE**)_2_ as an electrocatalyst was investigated. A Pourbaix diagram was generated by plotting data from DPVs measured under different pH conditions, from pH 6.6 to 8.0 (Figure 6b). This diagram shows that the oxidation wave of Cu^II^/Cu^I^ at +0.25 V is independent of the pH and is nonrelated to the catalytic event. At high potential, in which water oxidation takes place, a linear slope value of 0.055 was obtained at +1.37 V. This slope value is similar to the theoretical slope value of 0.059, which is in line with a PCET (nH^+^/ne^–^) process.^42^ Based on the Pourbaix diagram, and taking into account that the H_2_O bridge between the two Cu centers of Cu_2_(**BDiE**)_2_ is labile, we propose that Cu_2_(**BDiE**)_2_ is oxidized from Cu^II^-(H_2_O)-Cu^II^ to Cu^III^(OH)Cu^III^(OH) (**int1**, Figure 7), through subsequent PCET processes, where the bridging water molecule is deprotonated and dissociated from one of the Cu centers and a water molecule from the bulk solution coordinates to this Cu center and deprotonated with coupled-electron transfer.^27^ As the two Cu centers are symmetrical, we can suggest that in the next step, one of the Cu^III^–OH centers in **int1** is further oxidized by a PCET reaction to form Cu^III^(O^•^)Cu^III^(OH), **int2**.^40^ Once a reactive Cu-oxyl radical species is formed, it is stabilized by coupling with the coordinated hydroxide of the unreacted Cu^III^–OH center, and in turn, the high oxidation state Cu^III^ is reduced by pulling an electron from the OH ligand, and the Cu–OH bond is cleaved, yielding the hydroperoxide species **int3**.^43^ To support the formation of the O–O bond, we wished to generate the peroxo intermediate first by chemical oxidation with H_2_O_2_, characterize the product by infrared spectroscopy (IR) analysis, and then compare this spectrum to the one obtained upon the electrochemical (CPE) oxidation of Cu_2_(**BDiE**)_2_.^26^ The complex was thus oxidized by either H_2_O_2_ or CPE and in both cases the IR spectra exhibited a transmittance at 883–889 cm^–1^, assigned to the O–O bond of Cu–OOH. A control IR spectrum of unoxidized Cu_2_(**BDiE**)_2_ did not show this signal (Figure S16).^44−48^ These results support the formation of hydroperoxo intermediates during electrocatalytic water oxidation, which is consistent with the proposed **int3**. Finally, **int3** undergoes a further PCET reaction, forming and releasing O_2_, completing the OER cycle, and regenerating the initial species Cu_2_(**BDiE**)_2_. This pathway is further supported by the kinetic isotope effect (KIE) study in both H_2_O and D_2_O. The KIE value remains below 1.5 (from ∼1.1 to ∼1.4), regardless of the concentration of the catalyst (Figure S15), indicating that no atomic proton transfer process occurs during O–O bond formation.^19^ This proposed mechanism suggests that the diol side chains assist the H^+^ transfer during PCET processes, by doubly stabilizing them both in the first and second steps of the reaction, therefore facilitating the formation of **int2** for O–O bond formation.^49^

Although our results suggest the above pathway as the major mechanism of the reaction, they also indicate that *k*_B_ of borate buffer is not so low to be considered negligible but 0.88 M^–1^ s^–1^ and the KIE value is not 1 but ranges from 1.1 to 1.4. Hence, we propose that a minor pathway of the formation of the O–O bond involving borate buffer for proton transfer coexists within this reaction. In this pathway, instead of a H_2_O molecule from the blank solution participating in subsequent PCET reactions in the first step of the reaction, the Cu_2_(**BDiE**)_2_ undergoes the PCET processes followed by the formation of **int4**. In the next step, water nucleophilic attack (WNA) on **int4**, assisted by borate buffer, facilitates the proton transfer, leading to the formation of hydroperoxo intermediate **int5**. This intermediate is similar to **int3** from the major pathway, proven to be a hydroperoxo species, but in **int5**, the Cu ion bound to the peroxo group is in the 2+ state while in **int3**, it is in the 3+ state. Eventually, **int5** releases dioxygen and reforms back to Cu_2_(**BDiE**)_2_.

To support the suggestion that **int5**, which consists of Cu^II^–OOH, is a minor intermediate, the UV–vis spectrum was measured during the oxidation reaction both with H_2_O_2_ and by CPE. The UV–vis spectrum measured after the reaction with H_2_O_2_ showed that the absorbance at 320 nm is almost unchanged and only slightly increased (Figure S17). This result is different from the spectrum recorded during the CPE experiment showing a large decrease in the intensity at the same wavelength (Figure 4a).^26,27^ Our group has previously studied changes in similar absorbance spectra and concluded that such a decrease in absorbance is due to the oxidation of Cu^II^ to Cu^III^. As the oxidation in the reaction catalyzed here occurs electrochemically, it is reasonable that **int3**, which contains Cu^III^–OOH, is more dominant than **int5** having Cu^II^–OOH.^50^ These results further support the pathway involving hydroperoxo intermediate **int5** (Cu^II^–OOH) as a minor mechanism for the catalytic reaction, which coexists with the major mechanism where **int3** (Cu^III^–OOH) is dominant.

## 4 Conclusions

We introduced here the highly stable dinuclear Cu-peptoid, Cu_2_(**BDiE**)_2_, as an electrocatalytic OER at pH 7. The electrochemical properties, the OER performance, the buffer effect, and the benefits of peptoid ligand design have been investigated and discussed. Cu_2_(**BDiE**)_2_ can activate OER at pH 7 without buffer assistance, and more importantly, the diol side chains in the second coordination sphere accelerate the PCET reactions that lead to O–O bond formation and oxygen evolution. Along with this mechanism, a minor pathway involving a weak borate buffer effect for proton transfer during water nucleophilic attack is also proposed. Our study highlights the advancement in ligand design with the second coordination sphere effect for OER at neutral pH conditions and offers insights into protein mimics to address the oxidative transformation of small molecules.

## Supporting Information Available

Analytical HPLCs, ESI-MS, and ^1^H NMR spectra for ligands and complexes; cyclic voltammetry, UV–vis spectroscopies, IR spectra, EPR, and details of crystal structures for complexes; SEM and EDS for the surface of the electrode (PDF)
