# Supplementary material for: Biomimetic Second Coordination Sphere Effect within Cu-Peptoid Electrocatalyst Enables Homogeneous Water Oxidation at pH 7
Source: Inorg Chem. 2025 Feb 23;64(9):4267–74. doi: 10.1021/acs.inorgchem.4c04501 (PMC11898173; doi:10.1021/acs.inorgchem.4c04501)
Supplement: Supplementary file 1 — ic4c04501_si_001.pdf [file ic4c04501_si_001.pdf]

Supporting Information

Biomimetic Second Coordination Sphere Effect within Cu-Peptoid Electrocatalyst Enables Homogeneous Water Oxidation at pH 7

Guilin Ruan[a], Suraj Pahar,[a] Natalia Fridman[a], and Galia Maayan[a*]

[a] Schulich Faculty of Chemistry, Technion-Israel Institute of Technology, Technion City, Haifa 3200008, Israel. E-mail address: gm92@technion.ac.il.

Supporting Figures and Tables:

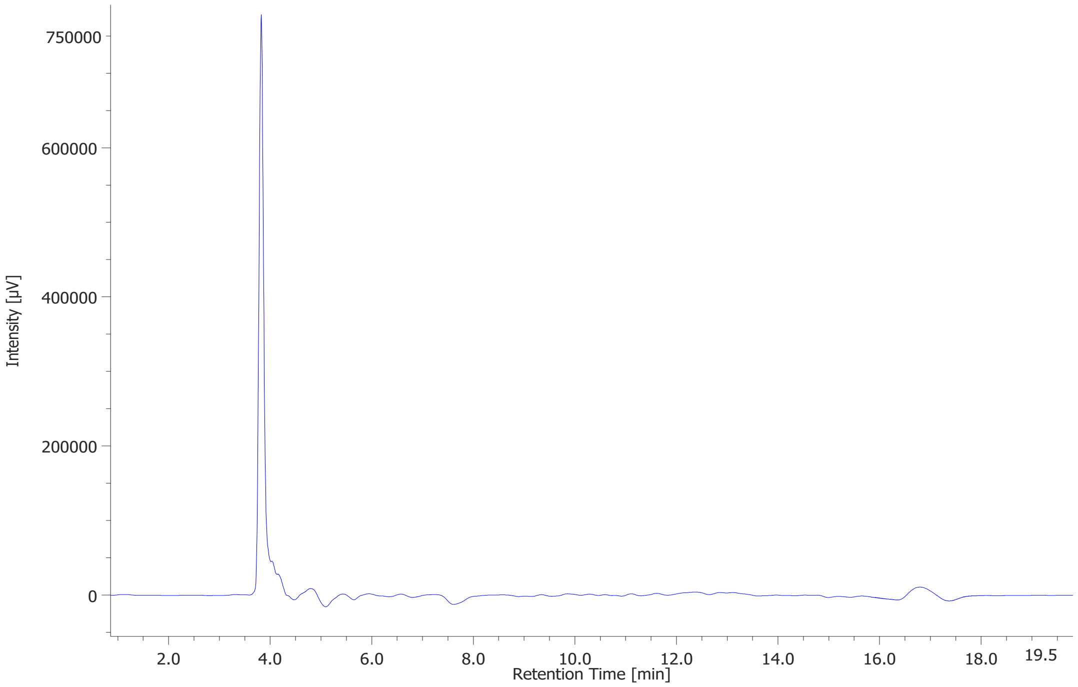

Fig. S1 Analytical HPLC of purified peptoid **BDiE** in water/acetonitrile with 0.1% TFA.

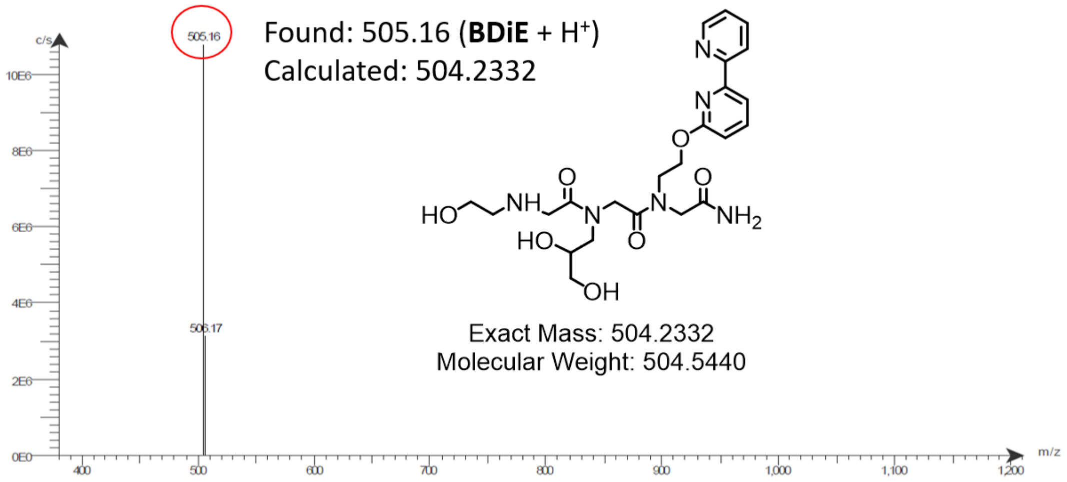

Fig. S2 ESI-MS of purified peptoid **BDiE** in water, m/z (**BDiE** + H^+) = 505.16.

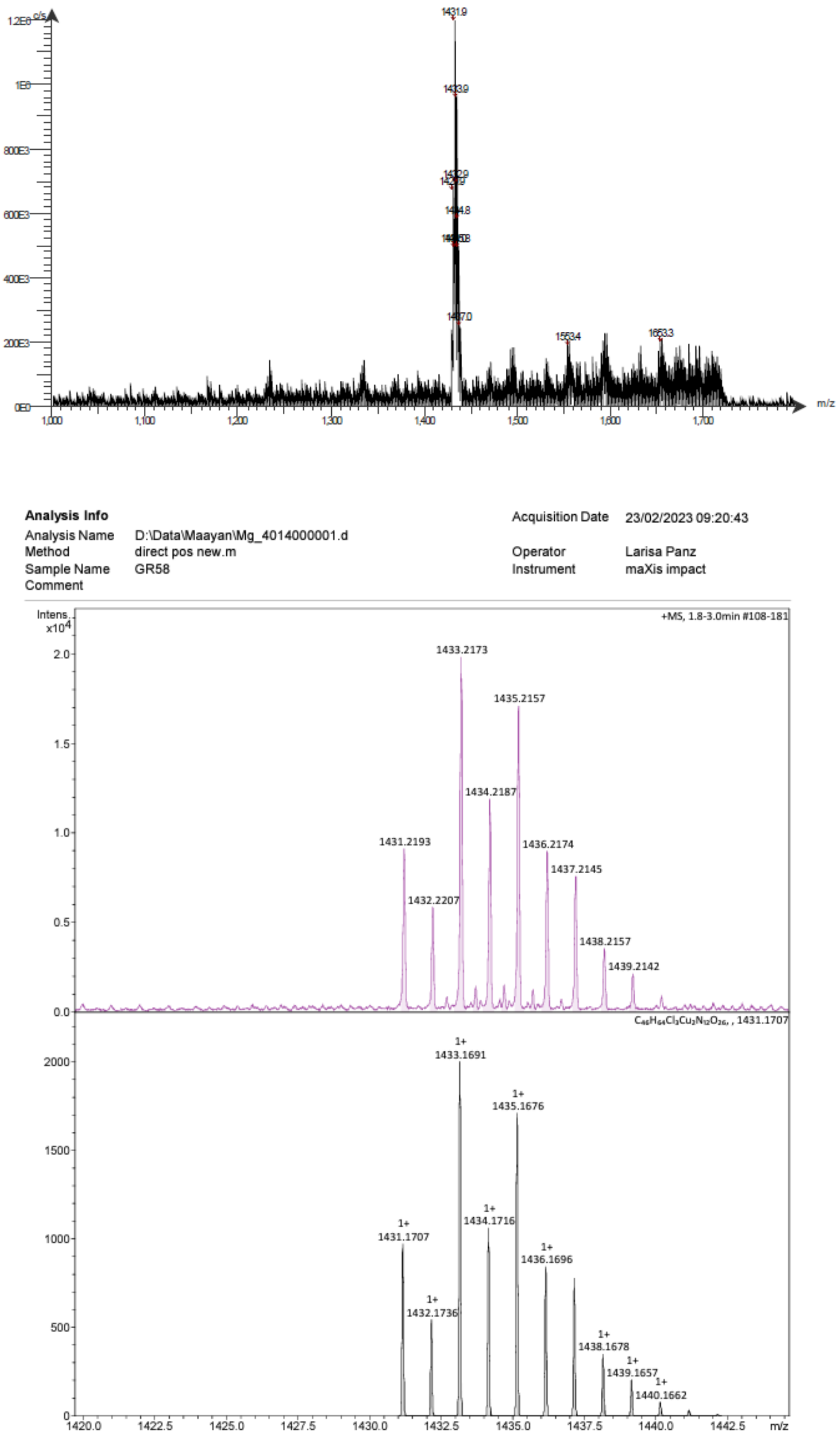

Fig. S3 Top: ESI-MS of Cu_2(**BDiE**)$_2$ in a wide range windows; bottom: experimental and simulated mass of [Cu_2(**BDiE**)$_2$(ClO_4^-)$_3$] = 1433.2173.

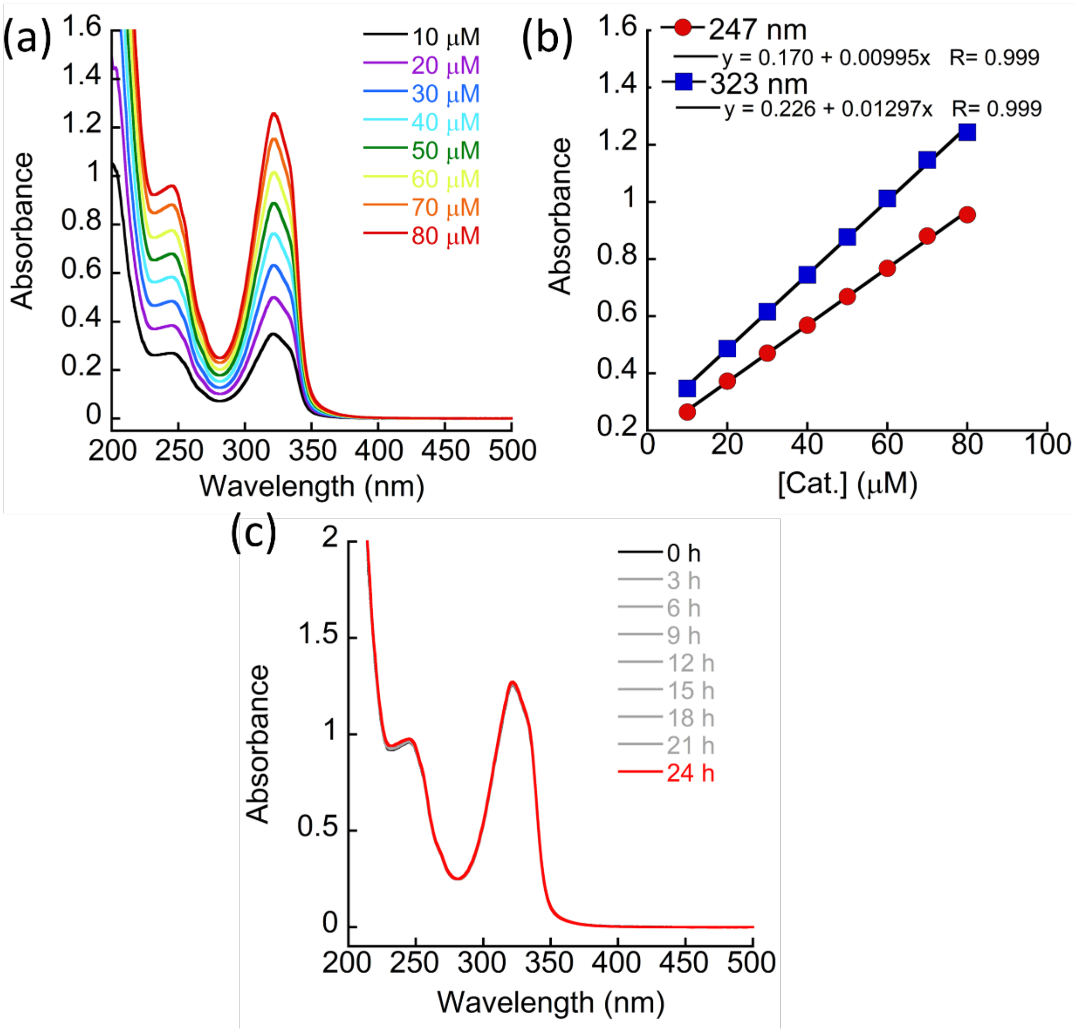

Fig. S4 (a) UV-vis spectra of different concentrations of Cu_2(**BDiE**)$_2$ in 0.2 M borate buffer at pH 7; (b) linear plots of the absorbance peak at 323 nm and 247 nm from the figure (a). (c) UV-vis spectra of Cu_2(**BDiE**)$_2$ in 0.2 M borate buffer at pH 7 for 24 hours.

Table S1. Bond-valence sum calculation parameters.

	**ro**	**rij**	**B**	**sij**	**z**
O3	1.655	1.995	0.37	0.401112	2.024753
O2	1.655	2.318	0.37	0.167096	
O6	1.655	2.478	0.37	0.106689	
N1	1.713	1.974	0.37	0.496585	
N2	1.713	2.047	0.37	0.408773	
N3	1.713	2.014	0.37	0.444498	

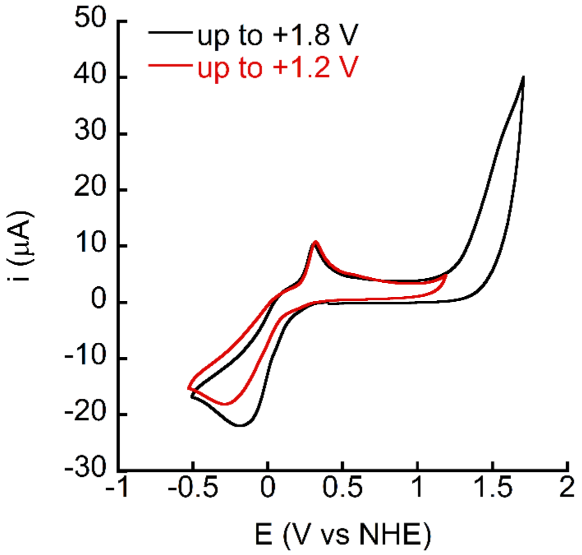

Fig. S5 Two CVs of Cu_2(**BDiE**)$_2$ in 0.2 M borate buffer pH 7 up to +1.2 V and +1.7 V. the difference at about -0.2 V indicates the oxygen reduction reaction with Cu^{II}/Cu^{I} reduction.

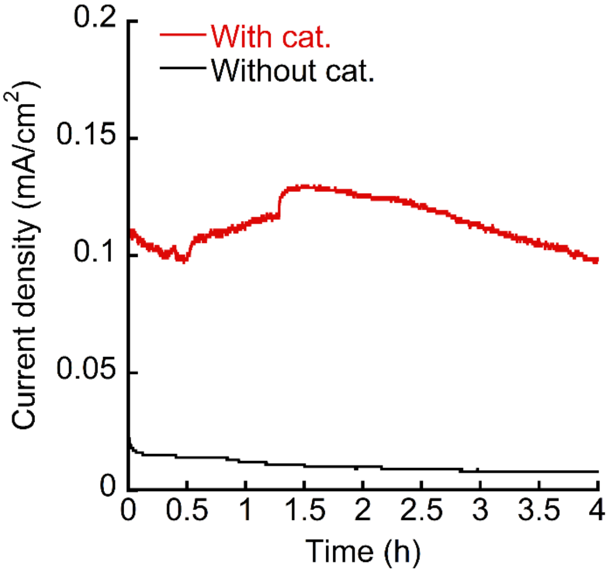

Fig. S6 Current density of Cu_2(**BDiE**)$_2$ and blank solution from 4-hour CPE experiment (as shown in Fig. 3) at applied potential +1.5 V vs. NHE in 0.2 M borate buffer at pH 7 using ITO as working electrode.

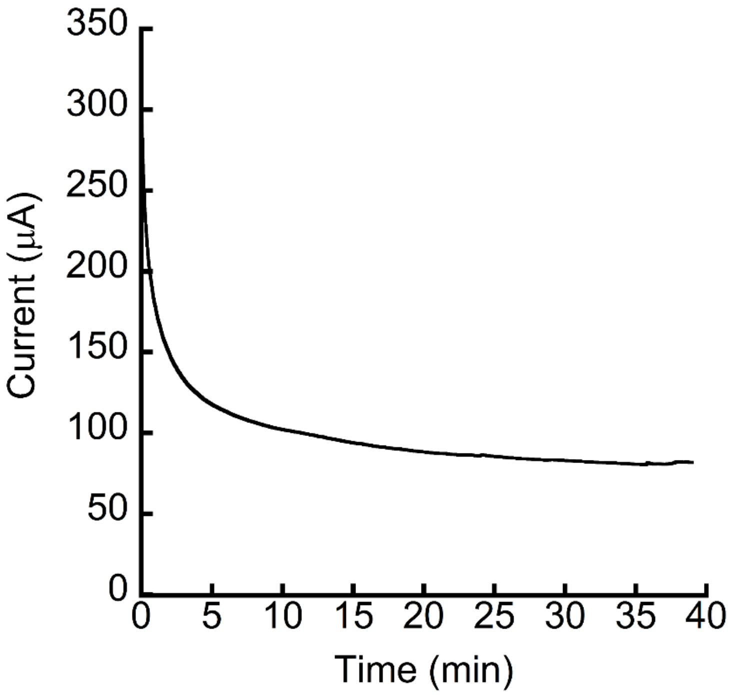

Fig. S7 Current density of Cu_2(**BDiE**)$_2$ from the 40-min spectroelectrochemistry experiment (Fig. 4) at applied potential +1.5 V vs. NHE in 0.2 M borate buffer at pH 7 using Pt net as working electrode.

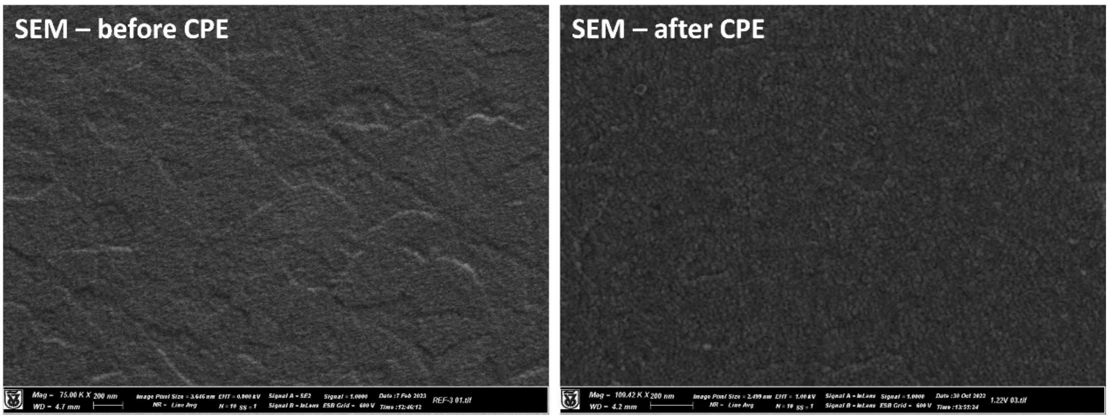

Fig. S8 HR-SEM image of fresh ITO electrode marked as “SEM-before CPE” on the left side and HR-SEM image of ITO electrode after 4-hour CPE experiment marked as “SEM-after CPE” on the right side.

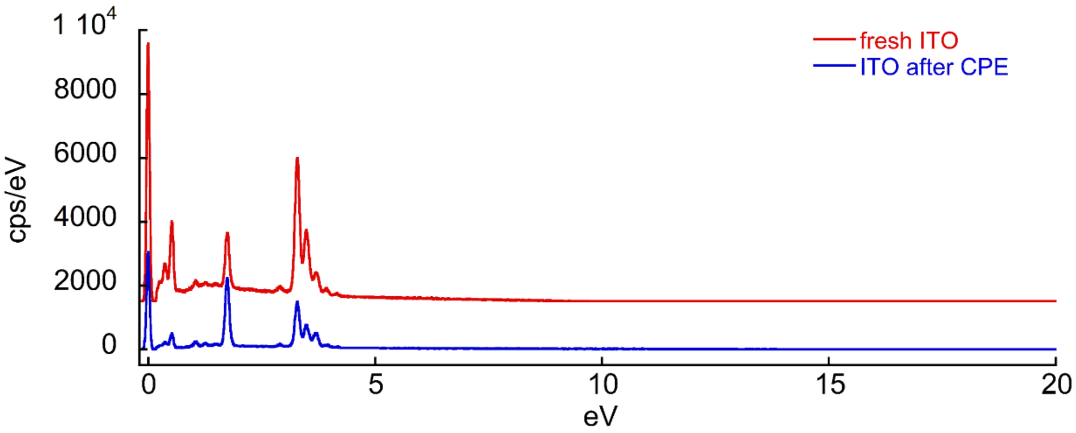

Fig. S9 EDX analysis of fresh ITO electrode (red) and ITO electrode (blue) after 4-hour electrolysis, no Cu element was detected at ~8 keV (K_α) and ~9 keV (K_β). For confirmation of sample composition, an acceleration voltage of 10 kV (from 1kV) was used. This higher acceleration voltage was needed for sufficient X-ray emission from the specimens.

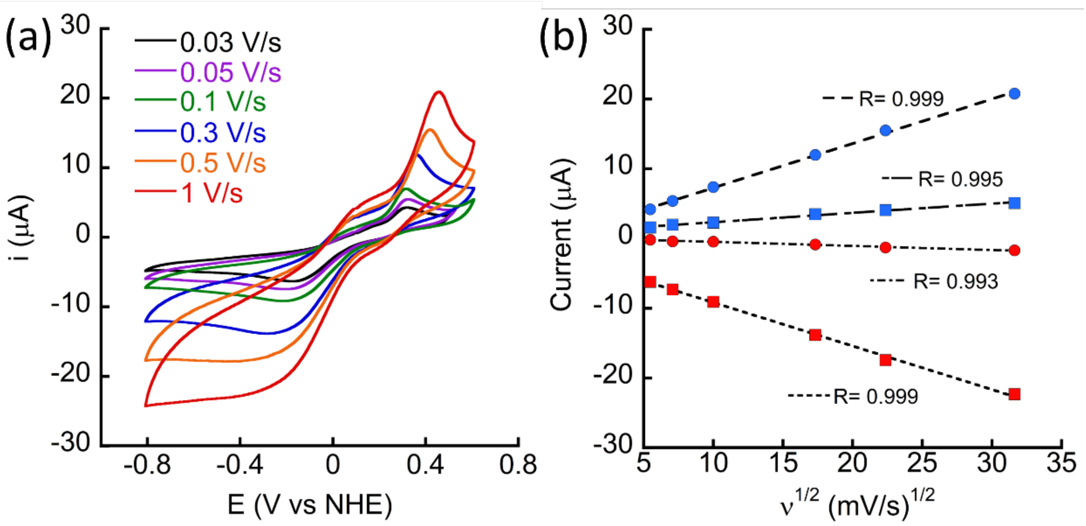

Fig. S10 (a) CVs of 0.5 mM Cu_2(**BDiE**)$_2$ at different scan rates with a narrow scanning range (-0.8 to 0.6 V vs NHE); (b) the linear regression of i_d current intensities versus $v^{1/2}$.

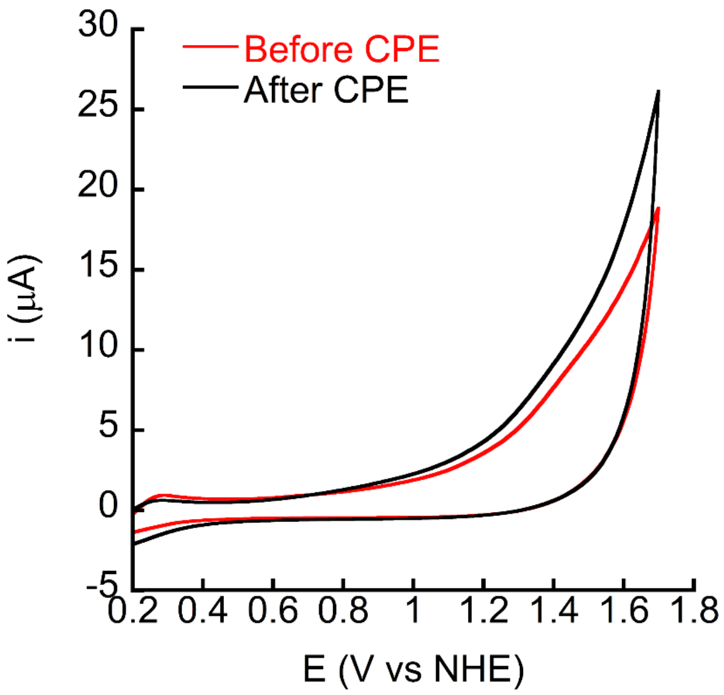

Fig. S11 CV scans of before and after 4-hour CPE experiment (shown in Fig. 3) of 0.25 mM Cu_2(**BDiE**)$_2$ in 0.2 M borate buffer solution at pH 7.

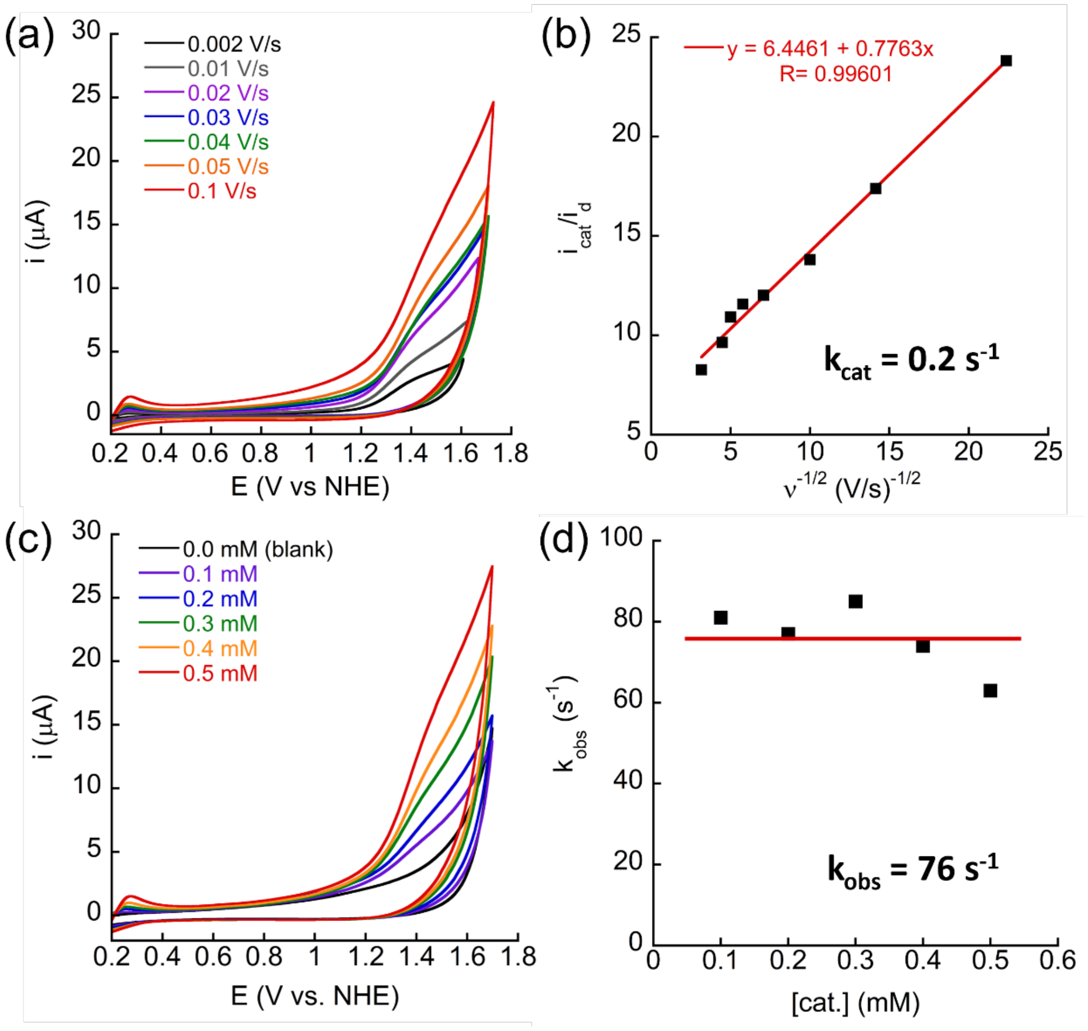

Fig. S12 (a) CVs of 0.5 mM Cu_2(**BDiE**)$_2$ at different scan rates in 0.2 M borate buffer at pH 7; (b) linear plot of i_{cat}/i_d vs. $v^{-1/2}$ using data from Fig. S12a and eq. (3); (c) CVs of Cu_2(**BDiE**)$_2$ with different concentrations in 0.2 M borate buffer at pH 7 at scan rate 100 mV/s; (d) calculation of TOF (k_{obs}) using FOWA method and data from Fig. S12c.

**The average k_{obs} was determined as 76 s^{-1} importing data of Fig. S12c to equation of FOWA below:*

$i_{cat}/i_p = 2.24n(RTk_{obs}/Fv)^{1/2}/\{1+\exp[F(E_{cat}-E)/RT]\}$

i_{cat} is the catalytic current in presence substrate (i.e. H2O);

i_d is the diffusion current without substrate (here the current is extracted from the redox event Cu^I to Cu^{II} at about 0.24 V as i_d as an approximation);

R is the universal gas constant;

T is the absolute temperature;

F is the Faraday constant;

v is the scan rate;

k_{obs}, which is also known as the turnover frequency (TOF), is the catalytic rate of the catalyst;

E_{cat} is catalytic potential;

E is the applied potential.

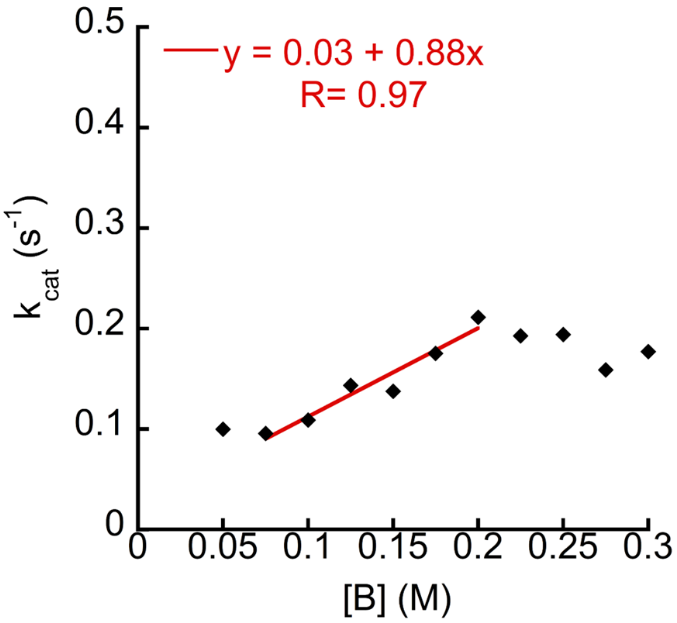

Fig. S13 The plot of kcat vs. concentration of borate buffer [B] in order to calculte the value of k_B following equation: $k_{cat} = k_{water} + k_B[B]$.

**From the result of Fig. S13, we observed that the overall data is non-linear, the linear relationship only showcases when the [B] increases from 0.075 to 0.2 M. The k_{cat} reaches a plateau when the [B] is increased higher. Therefore, buffer effect only shows up in this linear range. However, the calculated k_B is extremely low compared with other reported constant values, in other words, the buffer effect is extremely minor. Also, we believe that the k_{water} from the intercept value we obtained in the plot is not reliable because firstly, it has big error compared to the report k_{water}; secondly, we neglect the physical meaning of the flat data at low buffer concentration where there is no buffer effect while water is dominant for OER mechanism. If we consider the k_B of H_3BO_3 to reported k_{water}, we believe the buffer effect is negligible.*

Table S2. Summary the values of k_B and k_{water} from this work and previous studies.

	k_B (M^{-1} s^{-1})	k_{water} (s^{-1})	pH	Ref.
**H_3BO_3**	**0.88**	***0.03***	**7.0**	**This work**
$H_4BO_4^-$	750	0.42	9.0	[4]
HPO_4^{2-}	190	0.32	8.0	[5]
PO_4^{3-}	300	0.97	11.6	[6]

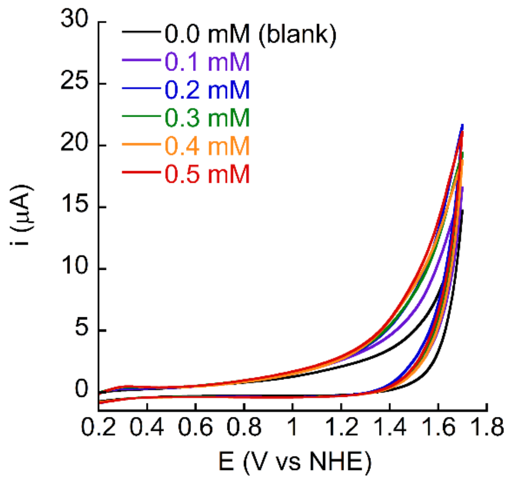

Fig. S14 CVs of different concentrations of the reported di-nuclear Cu peptoid [7] in 0.2 M borate buffer at pH 7 at scan rate 100 mV/s.

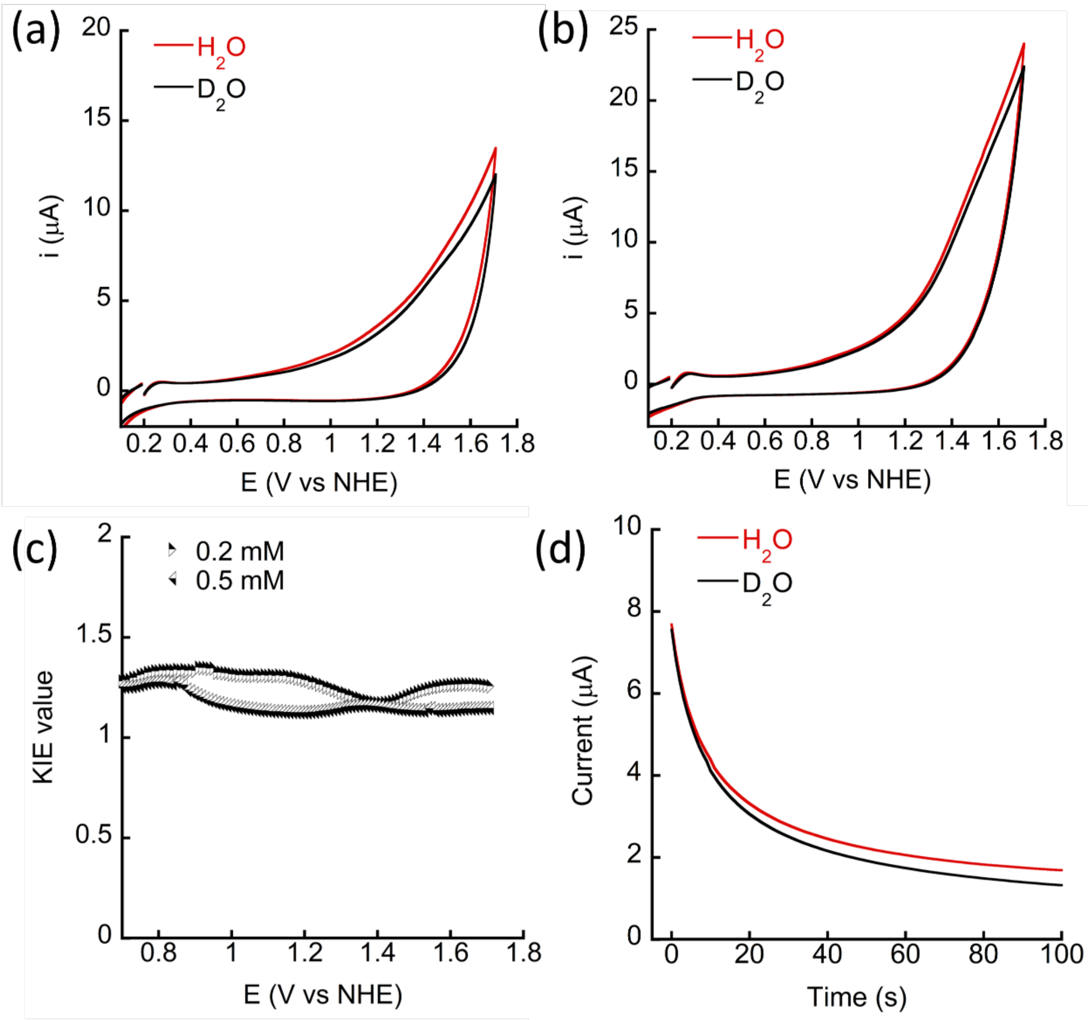

Fig. S15 CVs of (a) 0.2 mM or (b) 0.5 mM Cu_2(**BDiE**)$_2$ in 0.2 M borate buffer at pH 7 with H_2O or D_2O as solvent; (c) KIE values calculated by equation: KIE = $i(H_2O)^2/i(D_2O)^2$, each KIE value calculated with a pair of $i(H_2O)$ and $i(D_2O)$ at the same potential in Fig. S15a; (d) CPEs of 0.5 mM Cu_2(**BDiE**)$_2$ at applied potential +1.5 V in 0.2 M borate buffer with H_2O or D_2O as solvent using fixed surface area glassy carbon electrode (0.07 cm^2).

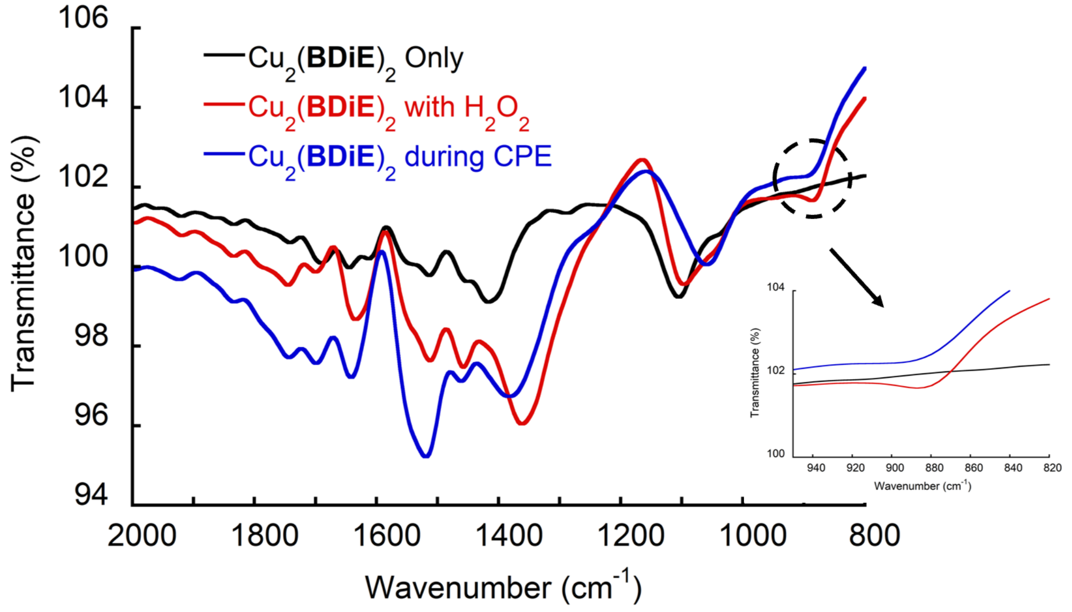

Fig. S16 FTIR spectra of the Cu_2(**BDiE**)$_2$ (1 mM) complex during electrolysis at 1.6 V vs. NHE and after the addition of a few drops of 2% H_2O_2 in 0.2 M Borate buffer pH 7.

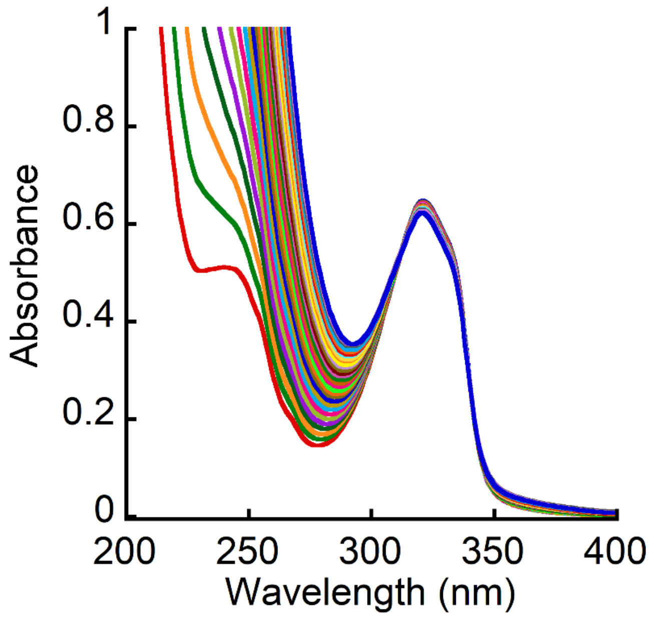

Fig. S17 Titration of ~30 μM Cu_2(**BDiE**)$_2$ with aliquots of 3 μL of 6% H_2O_2 followed by UV−vis spectroscopy in 0.2 M Borate buffer pH 7.

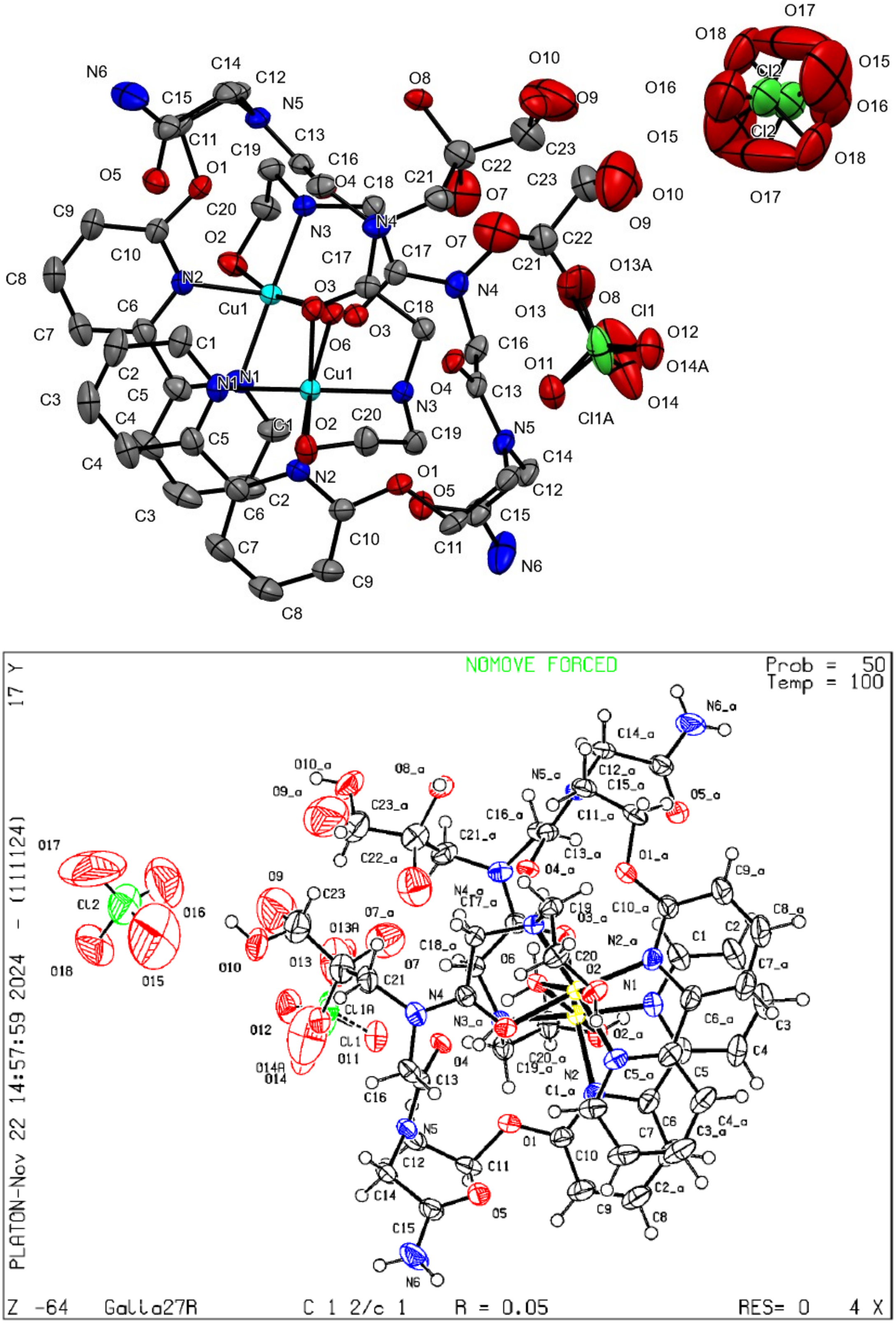

Fig. S18 Crystal structure of complex Cu_2(**BDiE**)$_2$ with atom labels, hydrogen atoms, and part of water guest molecules are omitted for clarity. Top figure from Mercury and down figure from the checkcif file.

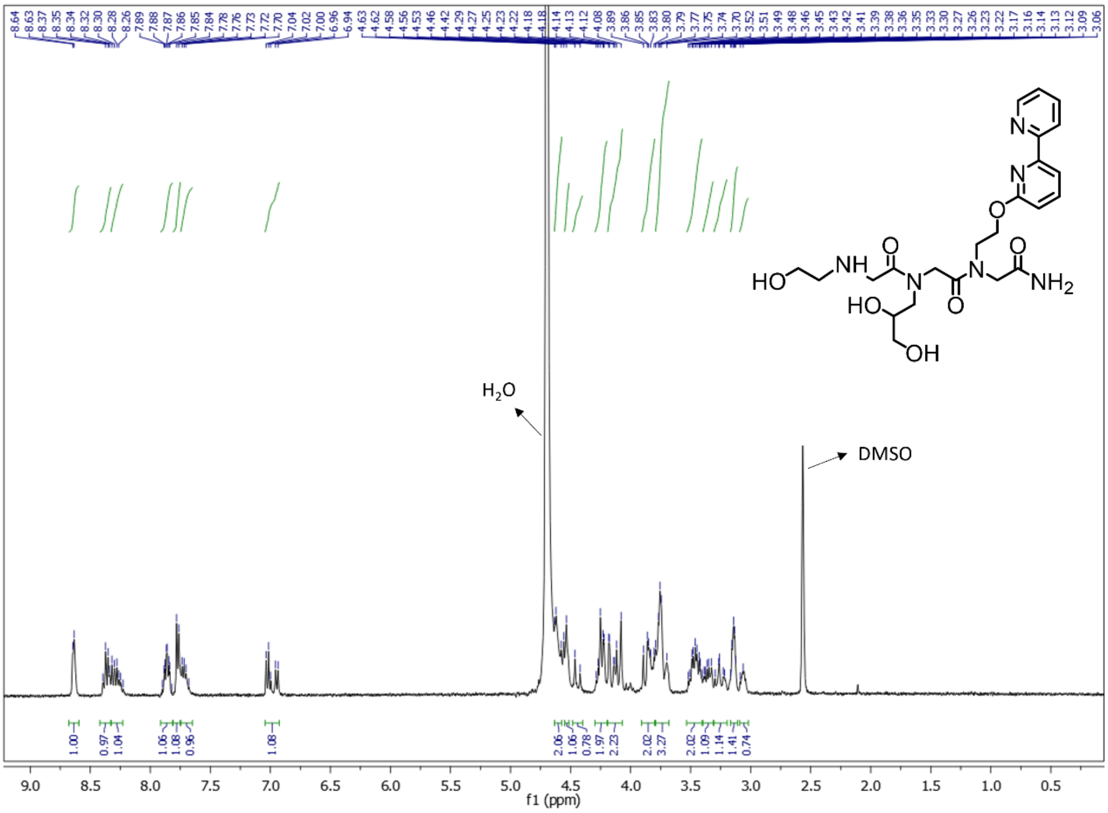

Fig. S19 ^{1}H-NMR (400 MHz, D_2O) of ligand BDiE. Assignment: aromatic H (δ 8.64 - 6.94, 7H), peptoid backbone -CH_2- and -CH- (δ 4.63- 3.06, 19H).

(a)

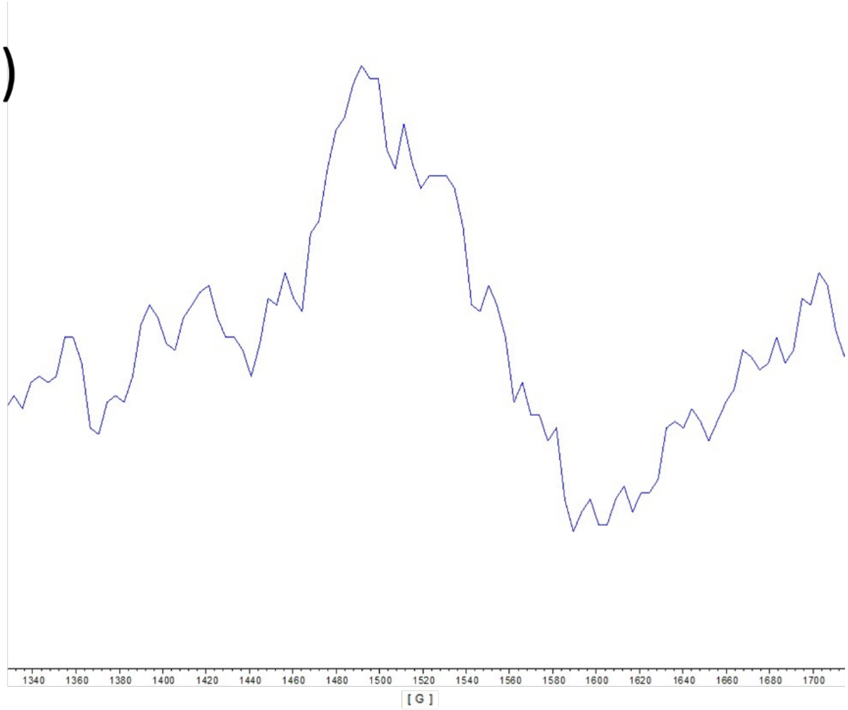

(b)

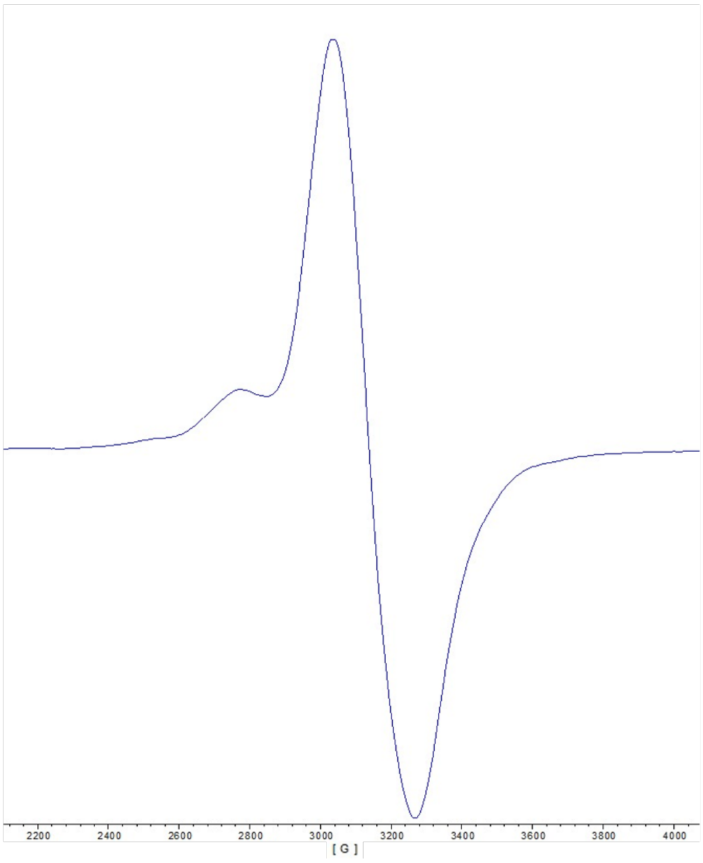

Fig. S20 (a) EPR spectra of Cu_2(**BDiE**)$_2$ at half-field region (ΔMS = ±2) from 1340 to 1700 G; (b) EPR spectra of Cu_2(**BDiE**)$_2$ at region (ΔMS = ±1) from 2200 to 4000 G; signal in (a) is 6-fold enlarged from (b); all the experiments were done at 200 K.

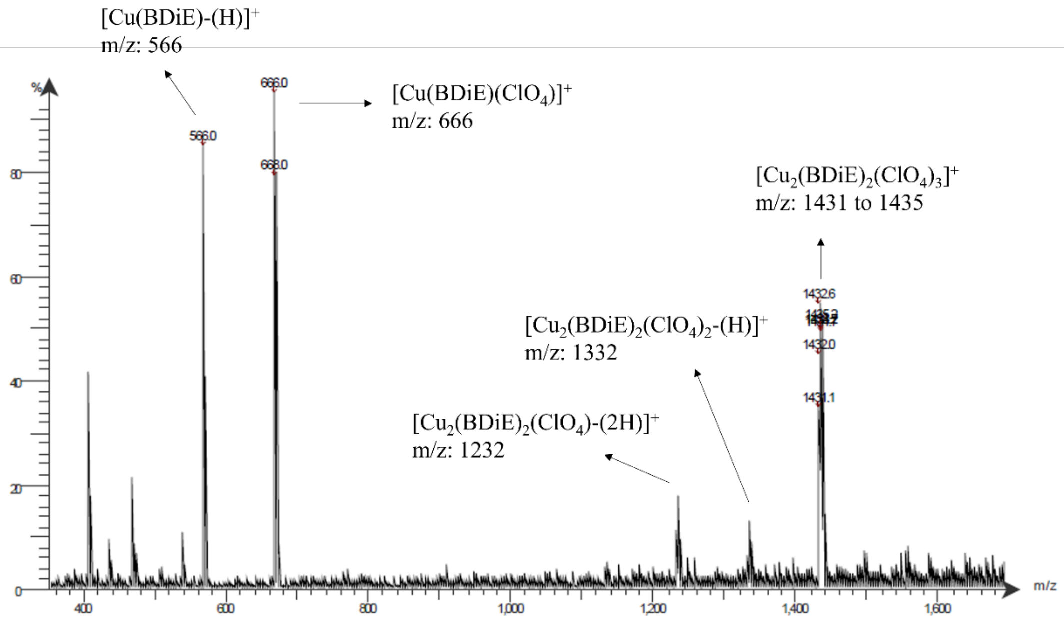

Fig. S21 Full ESI-MS spectrum of Cu_2(**BDiE**)$_2$; the observed peaks have been assigned besides those fragmenations below m/z 500, which have no Cu isotope.

Table S3. Crystal data and structure refinement for Cu_2(**BDiE**)$_2$.

Empirical formula	$C_{46}H_{61}Cl_3Cu_2N_{12}O_{27}$
Formula weight	1447.49
Temperature/K	100.15
Crystal system	monoclinic
Space group	C2/c
a/Å	16.5769(7)
b/Å	24.6926(8)
c/Å	18.4049(8)
α/°	90
β/°	114.700(5)
γ/°	90
Volume/Å^3	6844.4(5)
Z	4
ρ_{calc}g/cm^3	1.405
μ/mm^{-1}	0.823
F(000)	2984.0
Crystal size/mm^3	0.18 × 0.15 × 0.09
Radiation	MoKα (λ = 0.71073)
2Θ range for data collection/°	4.634 to 60.088
Index ranges	$-21 \leq h \leq 19$, $-29 \leq k \leq 32$, $-19 \leq l \leq 22$
Reflections collected	24412
Independent reflections	7531 [R_{int} = 0.0297, R_{sigma} = 0.0310]
Data/restraints/parameters	7531/498/483
Goodness-of-fit on F^2	1.071
Final R indexes [I>=2σ (I)]	R_1 = 0.0469, wR_2 = 0.1358
Final R indexes [all data]	R_1 = 0.0574, wR_2 = 0.1431

Table S4. Bond lengths for Cu_2(**BDiE**)$_2$.

Atom	Atom	Length/Å	Atom	Atom	Length/Å
Cu1	O2[1]	2.3182(19)	N5	C14	1.449(4)
Cu1	O3[1]	1.9952(18)	N6	C15	1.313(4)
Cu1	N1	1.974(2)	C1	C2	1.375(4)
Cu1	N2	2.047(2)	C2	C3	1.380(5)
Cu1	N3[1]	2.014(2)	C3	C4	1.378(5)
O1	C10	1.335(3)	C4	C5	1.389(4)
O1	C11	1.454(3)	C5	C6	1.478(4)
O2	Cu1[1]	2.3182(19)	C6	C7	1.379(4)
O2	C20	1.432(3)	C7	C8	1.381(5)
O3	Cu1[1]	1.9951(18)	C8	C9	1.374(5)
O3	C17	1.254(3)	C9	C10	1.395(4)
O4	C13	1.239(3)	C11	C12	1.504(4)
O5	C15	1.229(3)	C13	C16	1.528(4)
O7	O7[1]	1.700(13)	C14	C15	1.523(4)
O7	C22	1.527(7)	C17	C18	1.506(4)
O8	C22	1.464(5)	C19	C20	1.506(4)
O9	C23	1.335(10)	C21	C22	1.513(4)
O10	C23	1.397(6)	C22	C23	1.515(5)
N1	C1	1.340(4)	Cl1	O11	1.409(5)
N1	C5	1.344(4)	Cl1	O12	1.360(6)
N2	C6	1.356(3)	Cl1	O13	1.440(9)
N2	C10	1.340(4)	Cl1	O14	1.425(8)
N3	Cu1[1]	2.014(2)	Cl1A	O11	1.433(5)
N3	C18	1.479(3)	Cl1A	O12	1.447(5)
N3	C19	1.496(3)	Cl1A	O13A	1.437(8)
N4	C16	1.456(4)	Cl1A	O14A	1.386(10)
N4	C17	1.324(3)	Cl2	O15	1.475(9)
N4	C21	1.471(4)	Cl2	O16	1.4010(10)
N5	C12	1.468(4)	Cl2	O17	1.315(9)
N5	C13	1.346(3)	Cl2	O18	1.453(7)

Table S5. Bond Angles for Cu_2(**BDiE**)$_2$.

Atom	Atom	Atom	Angle/°	Atom	Atom	Atom	Angle/°
O3^1	Cu1	O2^1	92.77(7)	O1	C11	C12	106.4(2)
O3^1	Cu1	N2	174.00(8)	N5	C12	C11	113.9(2)
O3^1	Cu1	N3^1	82.81(8)	O4	C13	N5	122.6(2)
N1	Cu1	O2^1	92.45(8)	O4	C13	C16	121.2(2)
N1	Cu1	O3^1	92.98(8)	N5	C13	C16	116.2(2)
N1	Cu1	N2	81.38(9)	N5	C14	C15	113.3(2)
N1	Cu1	N3^1	171.29(9)	O5	C15	N6	124.0(3)
N2	Cu1	O2^1	89.53(8)	O5	C15	C14	122.0(2)
N3^1	Cu1	O2^1	80.19(7)	N6	C15	C14	114.0(3)
N3^1	Cu1	N2	103.05(9)	N4	C16	C13	111.3(2)
C10	O1	C11	119.4(2)	O3	C17	N4	120.9(2)
C20	O2	Cu1^1	105.94(15)	O3	C17	C18	119.5(2)
C17	O3	Cu1^1	113.57(16)	N4	C17	C18	119.6(2)
C22	O7	O7^1	155.0(4)	N3	C18	C17	110.2(2)
C1	N1	Cu1	125.2(2)	N3	C19	C20	111.4(2)
C1	N1	C5	119.9(2)	O2	C20	C19	106.4(2)
C5	N1	Cu1	114.47(19)	N4	C21	C22	112.8(2)
C6	N2	Cu1	111.74(19)	O8	C22	O7	119.6(3)
C10	N2	Cu1	128.74(17)	O8	C22	C21	105.7(3)
C10	N2	C6	118.5(2)	O8	C22	C23	109.6(3)
C18	N3	Cu1^1	109.90(15)	C21	C22	O7	107.2(3)
C18	N3	C19	113.4(2)	C21	C22	C23	111.8(3)
C19	N3	Cu1^1	109.94(15)	C23	C22	O7	103.0(4)
C16	N4	C21	118.4(2)	O9	C23	O10	111.2(6)
C17	N4	C16	117.9(2)	O9	C23	C22	114.7(6)
C17	N4	C21	123.6(2)	O10	C23	C22	109.8(4)
C13	N5	C12	119.8(2)	O11	Cl1	O13	109.9(6)
C13	N5	C14	123.5(2)	O11	Cl1	O14	107.3(6)
C14	N5	C12	116.6(2)	O12	Cl1	O11	114.5(5)
N1	C1	C2	121.4(3)	O12	Cl1	O13	107.2(5)
C1	C2	C3	119.3(3)	O12	Cl1	O14	109.5(7)
C4	C3	C2	119.5(3)	O14	Cl1	O13	108.4(8)
C3	C4	C5	118.8(3)	O11	Cl1A	O12	107.9(4)
N1	C5	C4	121.2(3)	O11	Cl1A	O13A	106.7(6)
N1	C5	C6	115.5(2)	O13A	Cl1A	O12	107.5(5)
C4	C5	C6	123.4(3)	O14A	Cl1A	O11	113.2(9)
N2	C6	C5	114.6(2)	O14A	Cl1A	O12	109.4(9)
N2	C6	C7	121.4(3)	O14A	Cl1A	O13A	111.8(10)
C7	C6	C5	123.9(3)	O16	Cl2	O15	106.2(5)

Atom	Atom	Atom	Angle/°	Atom	Atom	Atom	Angle/°
C6	C7	C8	119.3(3)	O16	Cl2	O18	107.4(4)
C9	C8	C7	120.0(3)	O17	Cl2	O15	110.9(4)
C8	C9	C10	117.8(3)	O17	Cl2	O16	115.7(6)
O1	C10	N2	112.5(2)	O17	Cl2	O18	112.3(5)
O1	C10	C9	124.7(3)	O18	Cl2	O15	103.5(6)
N2	C10	C9	122.8(3)				

References

1. Baskin, M., Panz, L. & Maayan, G. Versatile ruthenium complexes based on 2,2′-bipyridine modified peptoids. *Chem. Commun.* **52**, 10350–10353 (2016).

2. Ghosh, T., Ghosh, P. & Maayan, G. A Copper-Peptoid as a Highly Stable, Efficient, and Reusable Homogeneous Water Oxidation Electrocatalyst. *ACS Catal.* **8**, 10631–10640 (2018).

3. Zuckermann, R. N., Kerr, J. M., Kent, S. B. H. & Moos, W. H. Efficient method for the preparation of peptoids [oligo(N-substituted glycines)] by submonomer solid-phase synthesis. *J. Am. Chem. Soc.* **114**, 10646–10647 (1992).

4. Ruan, G., Fridman, N. & Maayan, G. Borate Buffer as a Key Player in Cu-Based Homogeneous Electrocatalytic Water Oxidation. *Chemistry A European J* **28**, e202202407 (2022).

5. Coggins, M. K., Zhang, M., Chen, Z., Song, N. & Meyer, T. J. Single-Site Copper(II) Water Oxidation Electrocatalysis: Rate Enhancements with HPO_4^{2-} as a Proton Acceptor at pH 8. *Angew Chem Int Ed* **53**, 12226–12230 (2014).

6. Tamaki, Y., Vannucci, A. K., Dares, C. J., Binstead, R. A. & Meyer, T. J. One-Electron Activation of Water Oxidation Catalysis. *J. Am. Chem. Soc.* **136**, 6854–6857 (2014).

7. Ruan, G., Ghosh, P., Fridman, N. & Maayan, G. A Di-Copper-Peptoid in a Noninnocent Borate Buffer as a Fast Electrocatalyst for Homogeneous Water Oxidation with Low Overpotential. *J. Am. Chem. Soc.* **143**, 10614–10623 (2021).
